# Dynamic Responsive Formation of Nanostructured Fibers in a Hydrogel Network: A Molecular Dynamics Study

**DOI:** 10.3389/fchem.2020.00120

**Published:** 2020-02-26

**Authors:** Jan Zidek, Andrey Milchev, Josef Jancar

**Affiliations:** ^1^Advanced Polymers and Composites, Central European Institute of Technology (CEITEC), Brno University of Technology, Brno, Czechia; ^2^Institute of Physical Chemistry, Bulgarian Academy of Sciences, Sofia, Bulgaria; ^3^SCITEG, Brno, Czechia

**Keywords:** hydrogel, molecular dynamics, relaxation, deformation, nanostructured, network, self-assembly

## Abstract

In an effort to study natural fiber formation, such as, e.g., spider silk, we present a model, which is capable of forming biomimetic fibrillar nanostructure from a hydrogel micellar network. The latter consists of interacting atomic groups which form cores of micelles, and of flexible chains forming the shells of the micelles. Micelles are connected in a compact network by linearly stretched chains. The structural elements of the network can be transformed during deformation from micellar into fibrillary type and their evolution is found to depend significantly on strain rate. Our model suggests a set of conditions suitable for the formation of nanostructured fibrillar network. It demonstrates that a fibrillar structure is only formed upon sufficiently fast stretching while, in contrast, the micellar gel structure is preserved, if the material is pulled slowly. We illustrate this key aspect by a minimalistic model of only four chains as part of the whole network, which provides a detailed view on the mechanism of fibril formation. We conclude that such a simplified structure has similar functionality and is probably responsible for the formation of nano-structured molecular fibrils in natural materials.

## 1. Introduction

The arrangement of molecules in natural materials is assumed to be optimal for their efficient functioning, motivating thus the efforts to mimic the natural arrangement also in man-made materials. Natural materials comprise a sequence of atomic groups, assembled in hierarchical structure on the nanometer and supermolecular level. Tensile deformation plays a key role in the formation of biological materials, such as spider silk, where the final structure consists of nano-crystalline inclusions incorporated in the rubbery network. In fact, the drawing of fibers is frequently used in laboratory and in industry (Mondal et al., 2008; Peng C. A. et al., 2017), and has been recently improved by means of electrospinning (Svachova et al., 2016; Srivastava, 2017). The electrospinning provides stretching of material at significantly higher stretching rates than simple mechanical deformation. Wang and Hashimoto (2018) observed a correlation between stretching rate and orientation of molecules during the electrospinning. They performed electrospinning of poly-vinylalcohol from water solution and recorded the process by high speed camera. Observed were domains of fibrillar material and bulges from disordered material whereby a higher speed of spinning lead to a decrease of the amorphous bulge size. The nanostructural arrangement has thus clear relation to the strain rate. Generally, however, the formation of nanostructured fibrils is still poorly understood while modeling studies suggest, that the formation of fibrils is based on plastic deformation of polymers.

In what follows we demonstrate that the rate of stretching governs the ultimate structure and superior properties of such natural materials. We develop a model, which mimics the functionality of spider silk in a simplified form, illustrating the formation of nanostructured fibrillar domains (Zidek et al., 2016). The resulting fibrillar structure formed by tensile deformation persists even after the deforming force is removed. The formation mechanism itself can be controlled by the stretching rate. The system is a micellar hydrogel network with an initial atomistic configuration, which behaves differently upon quick or slow deformation as the analysis of the structural evolution during the stretching process shows. Owing to the adopted simplified model, the mechanism of fiber formation can be readily monitored, suggesting the possibility of generating and testing similar materials of desired structure in a laboratory.

Our basic assumption is that the stretching rate controls fiber formation as has been observed and described recently (Zidek et al., 2016). In order to address the question as to why fibrils are formed only under specific circumstances, we consider the simplest structure, where structural aspects of the transformation become clearly visible in contrast to more complex natural materials. A relevant property of such system is that of block copolymer material with constant block length, end-capped by some interacting groups as, e.g., copolymer poly-lactide/glycolide/PEG, end-capped by itaconic acid (Michlovska et al., 2016). Our model reveals how an orientationally ordered structure can be created from this type of materials. The principles of the model are based on the notion of bundling (Benetatos and Jho, 2016) albeit there is currently no general theory of bundling in polymers. Bundles are assemblies of aligned semiflexible macromolecules, which are separated from other bundles by an amorphous matrix, or by empty space and do not aggregate into a single infinite bundle containing all molecules. In a network with covalent and physical crosslinks the semistiff polymer chains are usually attracted by Van der Waals and electrostatic forces. An essential factor is the existence of high energy barriers in the system, preventing condensation of all molecules into a single fibril. For instance, the bundling of DNA proceeds by transformation from thoroidal to rod-like shape whereby stable bundles are created (as in our model) whenever loops of chains can be transformed into bundles of straight chains overcoming a certain energy barrier.

An important precursor of the nanostructured self assembly is the domain structure. Nilebäck et al. (2018) investigated the self assembly of two neat materials, which are present in the natural silk. The first material represents a 4-fold repetition of alternated polyalanine and glycine-rich regions (4Rep), and the second one itself is globular C-terminal (CT). Both neat components form a disordered material as does a physical mixture thereof. The self-assembly to the fibrillar structure has been observed only in the case when 4Rep is connected to CT. Our fibers are also composed from domains of soft micellar structure and interconnected segments of stretched chain.

**Figure 1:**
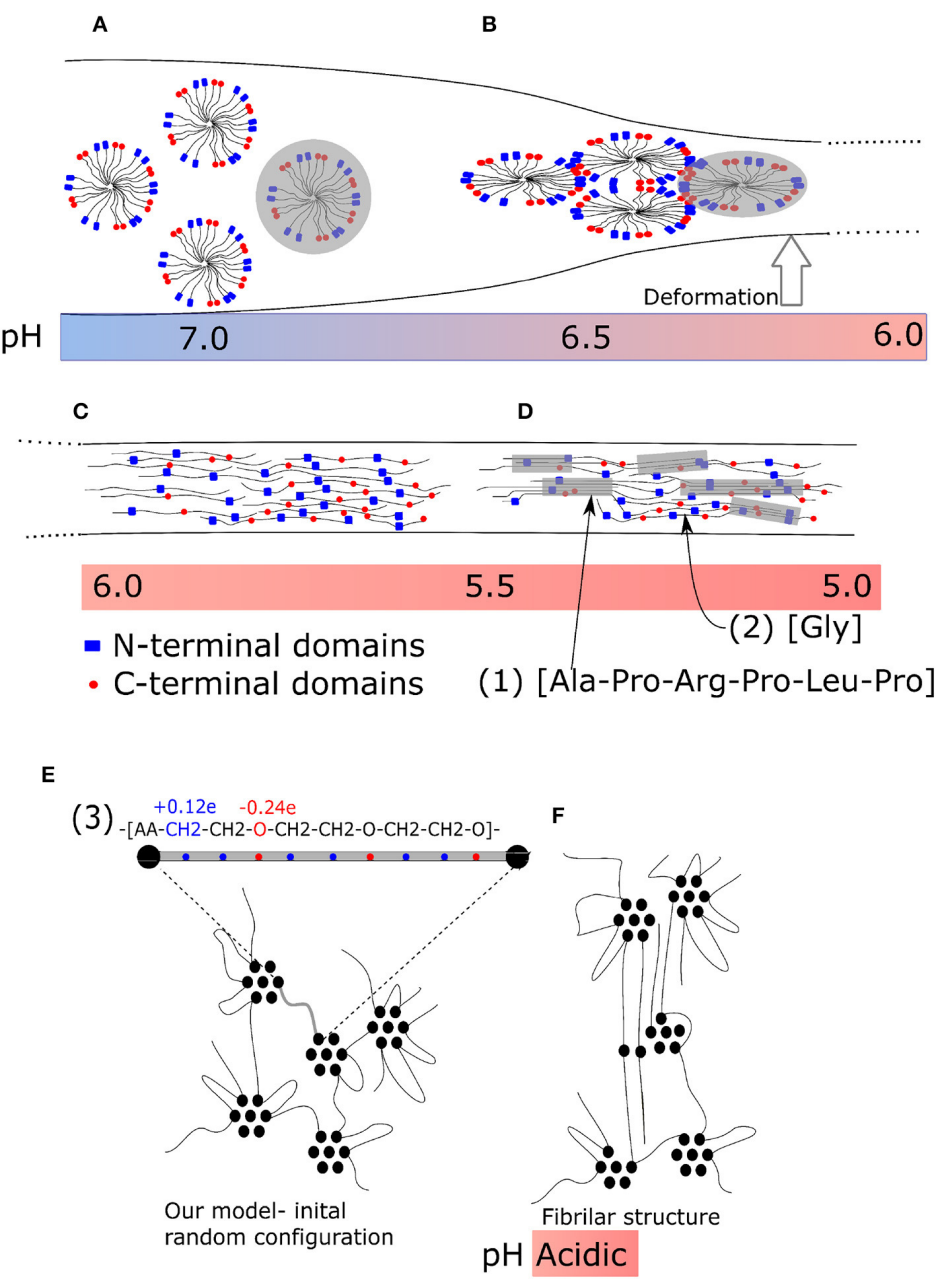
Fiber formation mechanism of spider silk during tensile deformation. **(A)** Micellar network: not deformed; **(B)** Liquid crystalline phase: moderate deformation; **(C)** Nanocrystalline fibrillar structure: strong deformation. **(A–C)** Enlarged parts of structures. (1) Typical secondary structure (amino acid sequence) of nanocrystalline chain, (2) typical flexible connecting chain rich in glycine, and (3) a repeating unit of our model. **(D)** Final structure of spider silk with β-sheets. **(E)** Micellar structure before deformation; **(F)** model nano-fibrillar structure after deformation.

An appropriate system, which can lead to bundles of molecules, is the solvated macromolecular network. An advantageous prerequisite situation thereby is a network that is swollen by the solvent. Several macromolecules can then stick together forming a fibril while the network does not collapse into a single fibril. That is probably the case of the spider silk. The original structure of spider silk fibroin is a micellar network (Jin and Kaplan, 2003; Walker et al., 2015) (Figure 1A). It is a liquid-like protein gel which remains liquid in the spider body, where deformation is slow, due to the micellar structure of the hydrogel. During quick extrusion, this gel is then transformed into a liquid crystal structure (Figure 1B), stretched chains (Figure 1C) and subsequently into β-sheets (Figure 1D). The initiating factor of the transformation is deformation of material *outside* the spider body.

The fibrils observed in spider silk (Wang et al., 2006; Perry et al., 2010) have well-defined molecular structure. Among the possible factors, which are believed to play a key role in self-assembly from the primary atomistic sequence, are exactly located interacting sites and side groups. Moreover, the transformation of a natural material is sensitive to a number of external conditions, such as *pH* (Figures 1A–D) and/or temperature. Originally, a micellar structure from silk spidroin is formed, followed by phase separation due to ion exchange acidification and water removal. During the second phase, a liquid crystalline phase is formed. Next, the pulling of fibers forms the final structure (Liu and Zhang, 2014). It has been found, that the stretching can induce hydrophobicity of the material (Lele et al., 2001) owing to cooperative hydrogen bonding of polymer chains. The detailed description morphology is provided by wide angle X-ray diffraction (Grubb and Jelinski, 1997). The liquid crystal transformation into final fiber is achieved by re-orientation during the stretching as an affine deformation of a network with inclusions.

Our model describes only mechanical transformation of the structure and does not include the chemical changes in the real spider silk fibers during formation. However, the induced hydrophobicity is observed as well. The energy of solvation is found to switch from negative to positive values. As a result, the stretching of the network leads to expulsion of water from the network.

The final structure of fibers is given by a specific sequence of amino acids that take part in the formation of β-sheets and sequences rich in poly-glycine [Figure 1D-sequence (1)], which form flexible chains. The sequences of β-sheets of nanocrystals must be aligned [Figure 1D-sequence (2)]. Also the end-to-end distance of a soft chain, rich in poly-glycine, connecting two β-sheets must be controlled. To some extent, the final structure of fiber material is encoded in the primary sequence of the amino acids. Also in our model the ability to form fibrillar structure is encoded in the alternating sequence of interacting acrylic acid (*AA*) and polyethylene glycol (PEG) molecules. The fibers of polyethylene contain domains of crystalline phase (Lee and Rutledge, 2011; Yamamoto, 2013), which alternate with large amorphous domains. The atomistic structure of spider silk and our model are micellar in the phase of geometry optimization.

The technique of thread drawing is an important aspect too, which determines the ultimate structure of the material. Andersson et al. (2017) considered laboratory drawing of silk spidroin and compared the laboratory-made and naturally spun fibers originating from exactly the same structure of natural silk fibroin so as to investigate the effect of fiber elongation. The laboratory spun fiber did not have exactly the same structure as the natural fiber. The principal property of spider silk, formation of β-sheets, was observed to a less extent in man-made fiber than in natural fiber. In contrast, fiber produced in laboratory contained rather helical ordering.

Another example of biological material formed by stretching is the fibronectin. This material contains cryptic sites (Zhong et al., 1998), which include disulfide bridges in particular position (Zhong et al., 1998; Peleg et al., 2012). The position of disulfide bridges is regulated by the *integrin* protein in molecular structure. The role of cryptic sites is to *align* the molecular structure, when it is stretched. In the fibronectin, the configuration of primary structure is an important factor too. We also found that the formation of intermolecular bridges has effect in our model. At present, we can only decrease the ability of fiber formation by the addition of random covalent bonds. When the concentration of covalent bonds exceeds a threshold, the fibrillar structure completely disappears (Zidek et al., 2016).

Molecular modeling is a powerful method for studying fiber formation by self-assembly of molecular structures, and several investigations have been carried out recently. The deformation and failure of self-assembled hydrogels were investigated by Hammond and Kamm (2008). They applied a coarse-grained model using molecular dynamics for the deformation of collagen bundles, which serve as building blocks of the oriented filament structure of collagen. Structural self-assembly from their precursor structure is frequently modeled in proteins. In particular, the secondary structure of spider silk was modeled recently (Gray et al., 2016). The irreversible formation of oriented blocks was observed also in swollen polymers (Miyazaki et al., 2006; Peng N. et al., 2017). As well, the strain-induced crystallization is typical for some biomaterials like the above mentioned spider silk (Peng C. A. et al., 2017), collagen fibrils (Buehler, 2006) or poly-lactides (Stoclet, 2016). In this work we demonstrate that the formation of fibrillar structure can be achieved from materials with very simple micellar structure (Figure 1E). The primary structure of our model appears favorable for the formation of bundles (Figure 1F). An essential factor thereby is the periodical sequence of constant blocks of poly(ethylene glycol) alternating with interacting groups of acrylic acid [Figure 1E-sequence (3)]. In principle, this is a common material, which does not have any special structure and needs no pre-ordering before stretching.

## 2. Models and Methods

### 2.1. Network Model

Fiber formation was investigated by utilizing two models: a network model and a four-chains model. Both models complement and comply with one another while each model provides different kind of information.

Our *network model* describes a rather general micellar structure, which serves as a prerequisite for the formation of fibrils. The network has been uniaxially stretched and the resulting structure was monitored during the stretching. Therefore, this model provides information about the response of a general network structure to tensile deformation. The system is a representative cubic box of a hydrogel network with 5 nm side size. The model is composed of 8 macromolecular chains with each chain comprising 200 molecular groups. The macromolecular phase is solvated by 2,447 molecules of water. The motion of atomic groups of chains and of the solvent and the deformation of the material were modeled by molecular dynamics simulation.

**Figure 2:**
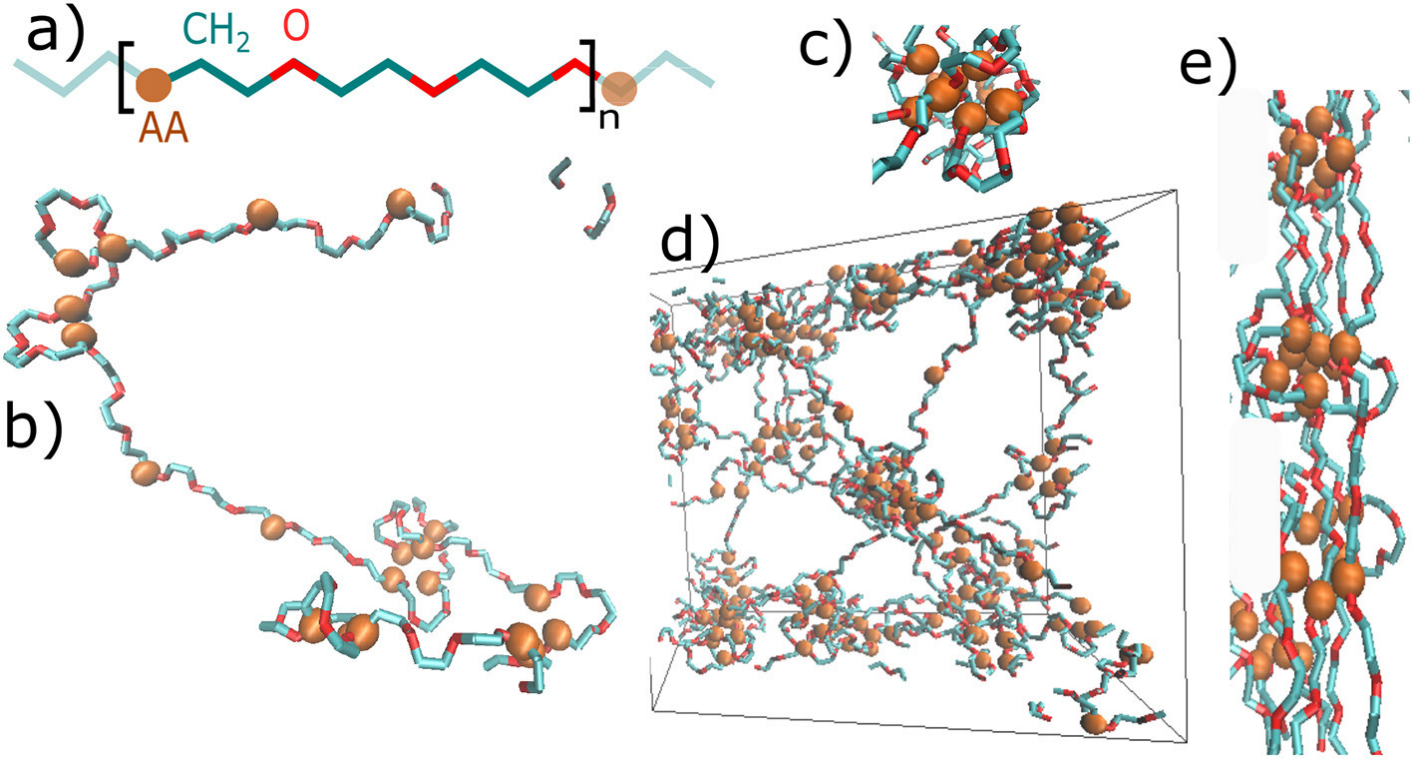
Snapshots of the hydrogel molecular structure: **(a)** primary structure—repeating unit of the chain: blue—methylene group of polyethylene glycol, red—oxygen of polyethylene glycol, *AA*—physically interacting group; **(b)** Secondary structure of a single chain: loops and stretched chains; **(c)** a single micelle; **(d)** dry phase of network: entire box (box side = 5*nm*); **(e)** fibril formed by deformation.

The structure of material is presented in Figure 2. Figure 2a shows the primary structure, which is based on a repeating unit of acrylic acid (*AA*), methylene group (*CH*_2_), and oxygen (*O*). Figures 2b–d show some structural elements, which have formed during the initial MD simulation. A single chain with given primary structure spontaneously forms loops and stretched chains (Figure 2b). The structure consists of cores of micelles, mostly made of *AA*-groups, and of flexible shells of micelles comprised of PEG chains. The micelles are organized into a micellar network (Figure 2d), as shown schematically in Figure 1D too.

The potential energy of the network was calculated from contribution of bonding energy, non-bonding Van der Waals interactions and electrostatic forces. The Hamiltonian of the simulation was calculated:

(1) $$\begin{array}{l} H=\underset{nb}{\sum }U_{b}+\underset{na}{\sum }U_{a}+\underset{np}{\sum }U_{el}+\underset{np}{\sum }U_{LJ} \end{array}$$

where nb is number of bonds, na is a number of bending angles and np is a number of non-bonding pairs. The energy of bonds and angles was calculated from harmonic potential, non-bonding interactions were taken as Lennard Jones potential function and the electrostatic potential was calculated from Coulomb electrostatic interaction potential. The details are presented in Supplementary Material.

The model was deformed at constant volume. The uniaxial stretching in z-direction was completed by compression in xy-directions. The model box has been stretched using seven different stretching rates. The rates are chosen in a logarithmic scale from 0.01 to 1 ns^−1^. In what follows we use the term “slow” for the deformation speed of 0.01 ns^−1^, and “quick” for 0.1 ns^−1^. The slow and quick deformations are found to induce significantly different network evolution. Therefore, we have examined the intermediate rates between slow and quick so as to get a better notion of the transition between both. Below, the rates are mostly referred to as a ratio to the quick deformation rate (Table 1).

**Table 1:** List of stretching rates (*r*) applied in this work.

**r[*ns*^−1^]**	**Word description**
0.01	Slow
0.02	0.2 × quick
0.05	Moderate, 0.5 × quick
0.1	Quick
0.2	2 × quick
0.5	5 × quick
1	Quickest, 10 × quick

These stretching rates are high in comparison to those used in real experiments. Molecular dynamics simulation deals with times in the range of hundreds of ns or μs. Stretching rates in classic deformation take approximately (10^−12^ *s*^−1^). The estimated stretching speed of electrospun material in the article of Wang and Hashimoto (2018), measured from image analysis of the high speed camera, was (10^−7^ *s*^−1^). Nevertheless, a clear qualitative correlation between stretching rate and the process of orientation was observed in our simulation too. The results for one stretching rate of network model were calculated from 4 independent simulations with different initial atomistic configuration.

The deformation of the system is reflected by the change in energy density, which can be transformed itself into a stress-strain relationship. The stress strain relationships are presented elsewhere (cf. Zidek et al., 2016, 2017b). We assume that the structure gets organized during the stretching and bundles form. The bundles are objects of three or more mutually aligned chains. An example of bundles is displayed in Figure 2e. Bundles were analyzed according to their orientation in space or depending on the degree of twisting (see the Supplementary Material). An object is identified as a bundle, when it satisfies several conditions:

- The bundle must be composed of 3 or more aligned short flexible chains.
- The end-to-end distance of all chains in the bundle between two physical clusters must be sufficiently long, >0.65 nm.
- The bundle must connect two physical clusters.

The next aspect of deformation is the damage of the physical network and its recovery with time. The damage affects the micellar structure of the network, displayed by the cores of micelles, which form *physical clusters* (PCLs) in the network. The strength of a physical network is proportional to the volume fraction of PCLs. Therefore, the damage can be estimated as a decrease of the volume fraction of PCLs. A structural recovery means that the structure after deformation has been restored to the original one (Figure 2d).

The volume fraction of physical clusters is calculated in this work by triangulation of space between the interacting groups, namely, by 3*D*-Delaunay triangulation (DT). DT is itself a mathematical algorithm, which creates a triangular 3-dimensional mesh from the interacting *AA*-groups in the cores of micelles. The distribution of connecting lines is related to the fraction of clusters. Detailed information about the modeling method and the parameters of the models are provided in Supplementary Material.

### 2.2. The Four Chains Model

A disadvantage of the network model is that one cannot specifically control the mutual position of particular chains, the combination of angles between stretched chains as well as their end-to-end distances, even though the network model shows the evolution of the entire network structure.

**Figure 3:**
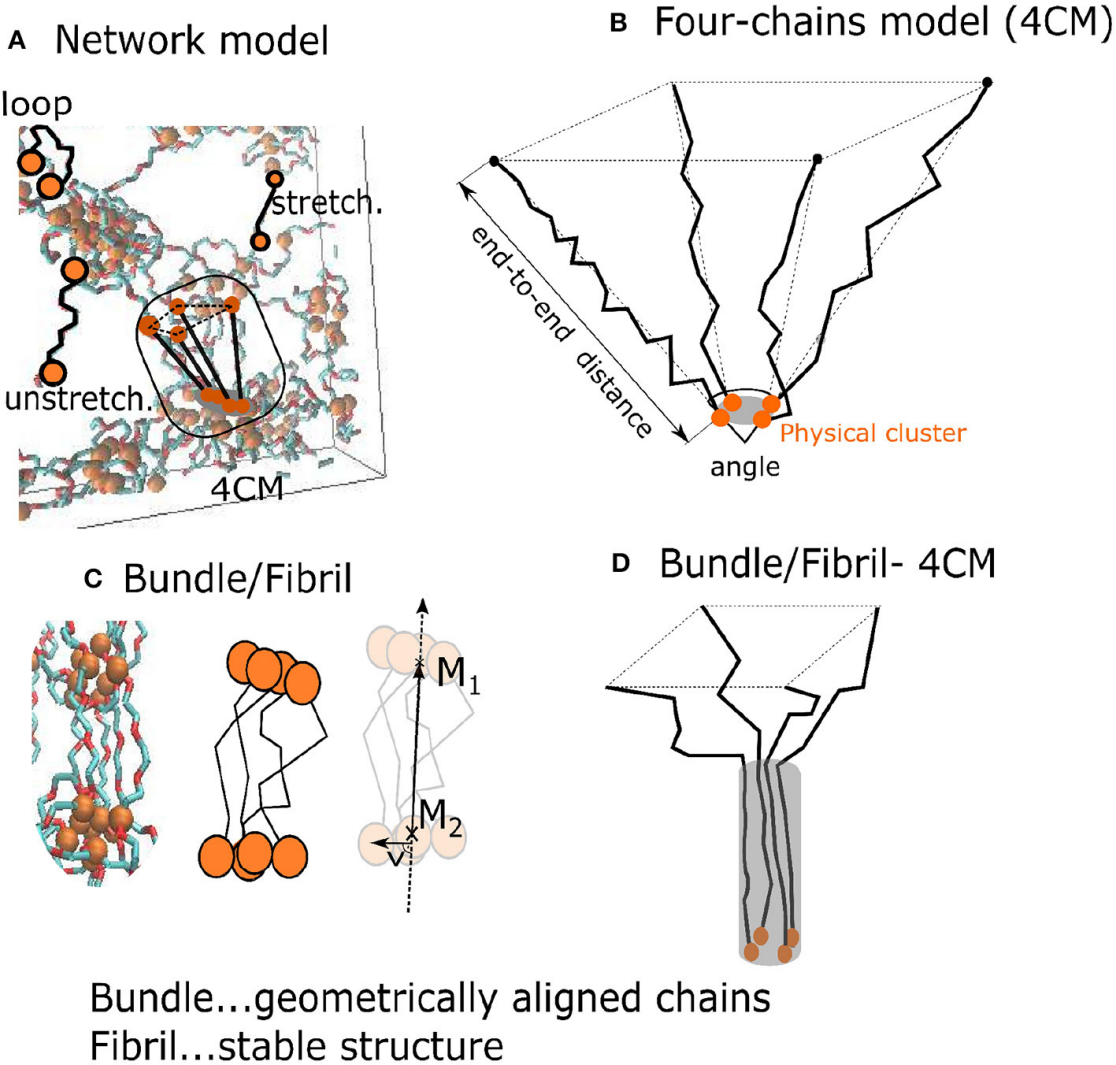
Two models of the hydrogel: **(A)** A network model with a subset of four 4 PEG chains around a physical crosslink; **(B)** “4-chains model”—four randomly generated freely rotating chains with known length and mutual angle. The orange disks are interacting *AA*-groups; **(C)** a fibril with a fiber vector $\vec{M_{1}M_{2}}$; **(D)** black lines denote chain segments aligned over some distance from the one end.

The auxiliary “4-chains model” is a minimalistic one, composed of only four flexible chains which span interacting groups. The four chains model is a structural element which is present also in the network model (Figure 3A). This simplified model controls the mutual orientation of chains and their end-to-end distances (Figure 3B). The composition of chains can be varied and one can analyze its influence on the formation of fibrils, which is not possible within the network model.

The bundles and fibrils were identified in the network model and in the four chains model (Figures 3C,D). We characterized the resulting fibril shape by means of the 2nd Legendre polynomial (*P*_2_), which describes the orientation of different segments (each segment standing for two consecutive bonds) of the chains. The orientation of a segment is determined with respect to the fiber vector. The latter is sum of the end-to-end vectors of all four chains. *P*_2_ describes the correlation of the fiber orientation with the individual segments of the chains, whereby *P*_2_ = −0.5, when the segments are oriented perpendicular the to fiber, *P*_2_ = 0, for randomly oriented segments, and *P*_2_ = 1, when all segments are parallel to the fiber:

(2) $$\begin{array}{l} P_{2}=0.5\left(3\langle \cos^{2}\theta \rangle -1\right), \end{array}$$

where θ is the angle of a segment with the fibril vector.

### 2.3. Relaxation of Network

The relaxation was analyzed immediately after the system was deformed. In fact, the relaxation contains several effects which occur during different time scales. That is why we performed three relaxation simulations lasting 1, 100, and 100,000 ps (100 ns) with the same initial configuration and initial random seed.

Each relaxation is characterized by specific correlation function, taken as the average of dot products of vectors in time 0 and *t*:

(3) $$\begin{array}{l} f\left(t\right)=\langle x\left(0\right)\cdot x\left(t\right)\rangle \end{array}$$

where *x* is a specific property at zero time and at time *t*. Observables are the inertia (velocity vector), bond/angle relaxation (bond vector), conformation relaxation (bond vector), relaxation of physical clusters (probability of presence in physical cluster) and relaxation of bundles and fibrils (bundle vector). Details are presented in Supplementary Material. The correlation functions were fitted to exponential decay.

## 3. Results

In our recent study (Zidek et al., 2017b) we described the deformation and macroscopic changes in a model structure of a hydrogel. We found that the stable fibrils appear, when the deformation is quick and sufficiently strong. The model revealed sensitivity to strain rate during deformation, i.e., to “slow” and “quick” (quick = 10× slow) deformation, starting with identical initial atomistic configurations, and external conditions, such as temperature. The qualitative difference in deformation behavior in both cases was related to damage in the primary micellar network whereby slow deformation leads to hydrogel network recovery while quick deformation was found to form permanent fibrillar structure that remained stable with time even against reverse deformation. Apparently, the structural recovery depends on the ability of the system to recreate spontaneously its original micellar structure.

### 3.1. Damage Recovery Within the General Network Model

**Figure 4:**
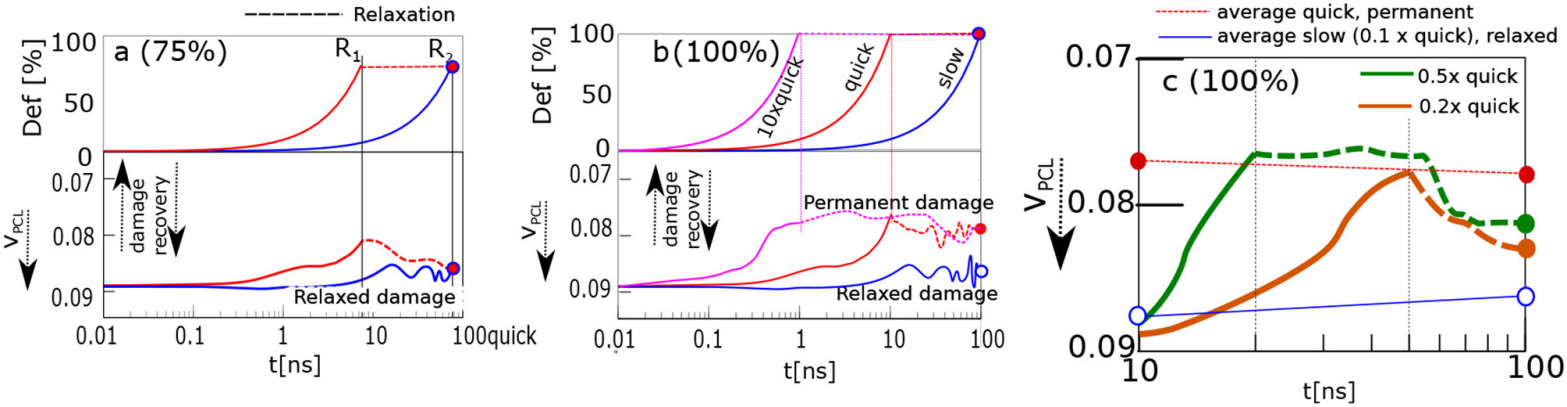
Damage recovery, when the sample is stretched and held in stretched state. Panels correspond to stretching ratios: **(a)** 75%; **(b)** 100%, “slow,” “quick,” 10 × “quick”; **(c)** 100%0.2× “quick,” 0.5× “quick.” Upper parts of **(a,b)** display stretching ratio, lower parts—damage, i.e., the inverse volume fraction of physical crosslinks, *V*_*PCL*_. Reference points with the same stretching ratio and simulation time, *R*_1_—blue, magenta curves, *R*_2_—all curves.

The recovery of the physical network was calculated by analyzing the time evolution of the volume fraction of physical clusters (cores of micelles, Figure 2c). The response of the material is recorded in Figure 4. The upper panel shows deformation in % as a function of time. The lower panel displays a reciprocal function of volume fraction of physical clusters. It describes the degree of physical network damage.

The model structure was slowly stretched in the computer experiment. The stretching rate was 100% deformation in 100 ns. The network was stretched up to 75%, and 100% of its original size. The course of slow deformation is shown as a blue curve. The network was stretched until the reference point *R*_2_. Alternatively, the same structure was stretched quickly by 10 times higher rate than the slow one: cf. red curve, Figure 4. The deformation was stopped at time *R*_1_. The simulation box was then held in a stretched state until the time *R*_2_. Thus, both samples were stretched effectively to the *same* stretching ratio within the same total time interval yet with a different deformation history.

The results from Figure 4a about the network stretched up to 75%, can be interpreted in terms of dynamic structural recovery. In that case the damage is a function of the stretching degree and rate.

Dynamic recovery is *not* observed in Figure 4b, when the network is stretched to 100% deformation. In that case, the quickly deformed network stays damaged. We analyzed different reasons, as to why the damage did not vanish. For example, we extended the time of relaxation to 1000*ns*. Alternatively, the network was compressed by reverse deformation to its original undeformed shape. None of those lead to a recovery.

We found permanent fibers (Figure 2e) in the network, which proved persistent. At temperature 300 *K*, the fibrils could not be removed in any way.

The “quick” and “slow” deformations are limiting cases. While the network relaxes during the “slow” deformation, during a “quick” deformation this is not the case so permanent transformations occur after the deformation. We analyzed the evolution of network after 2×, 5×, and 10× increase of the “quick” deformation rate. The fastest deformation (10× “quick”) is presented in Figure 4b. This fastest deformation leads to permanent changes similar to “quick” deformation.

Next we explore intermediate stretching rates between “slow” and “quick” in more detail. The first finding is, that there is ~50 ns period after the moderate deformation. During this period the damage persists before the onset of relaxation.

Another observation is that the network undergoes only partial relaxation (see Figure 4c), whereby the damage persists longer than the displayed interval of 100 ns as a longer simulation indicates. Probably, the lack of complete recovery is due to partial formation of fibrils.

It has been suggested in the literature, that the changes can be caused by solvent effect. It has been known that a similar effect of the small monomer and solvent molecules can play important role in the structure transition of the deformed networks (Warner and Terentjev, 1996). The water molecules are expelled which facilitates the alignment and bundling of fibers as the hydrogel is stretched. We investigated the solvation effect during the deformation of our network (Zidek et al., 2017a). It was found that the reconstruction of solvent may influence the deformation response only in the case of strong electrostatic interaction between polymer chains and water molecules as in the case of polyelectrolyte network. In the present network we use moderate electrostatic interaction between chains and water. In that case, one observes a reconstruction of the solvent. We found that solvent had only minor influence on mechanical response of polymers.

**Figure 5:**
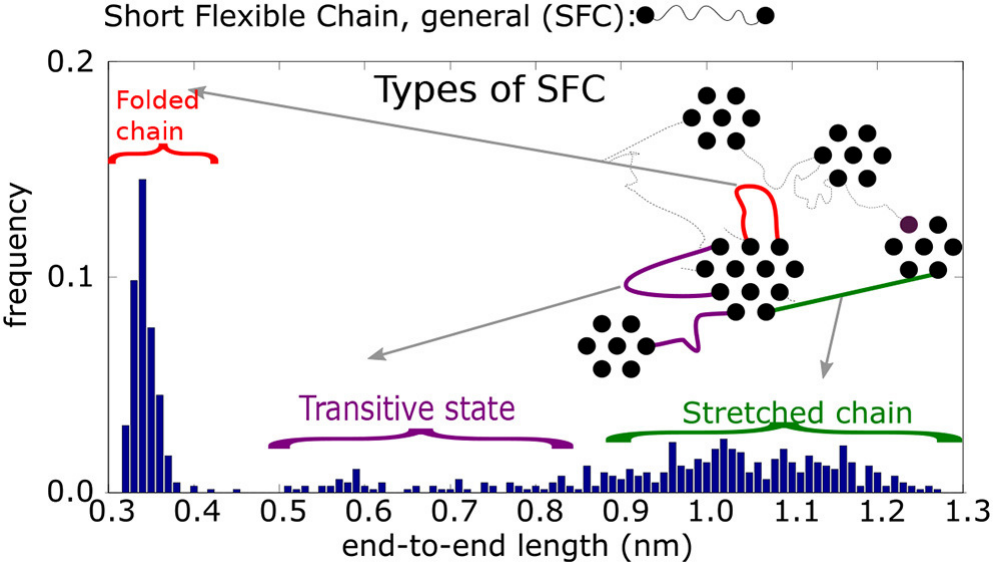
Histogram of end-to-end distances *r*_*e*_ of Short Flexible Chains. A SFC is the part of a chain between two physical crosslinks: schematic picture of four types of SFC and their end-to-end distances.

For the detailed structural analysis of the fiber formation mechanism we consider a structural component in the atomistic configuration, namely a short flexible chain, SFC (Figure 5). Such chains are parts of the macromolecular chain along with the end atomic groups of SFC, i.e., the *AA* (acrylic acid) groups. The positions of these interacting groups are fixed within the existing clusters. Three consecutive polyethylene glycol monomers (each monomer made of three atomic groups) separated by *AA*-groups define a SFC. In the present model, all SFCs consist of 11 segments.

Two *AA*-groups at both ends of a chain may belong to the same physical cluster so that this SFC is a loop. Alternatively, both chain-ends can be part of different clusters and the SFC is then a connecting chain. The motion of chain-ends is thus restricted inside, or between physical clusters, while the other segments of the chain can move freely in space. So in our model we deal with freely rotating chains with fixed chain ends and weak non-bonding interaction. Their motion in space is then determined by thermal displacements of the chains.

A histogram of end-to-end distances, *r*_*e*_, of unrestricted SFC should display a Gaussian peak. The actual end-to-end distance distribution, however, is more complex (cf. Figure 5). According to this histogram, the SFCs can be put into four classes, depending on their end-to-end distances, *r*_*e*_. In addition, we take into account the connectivity of physical clusters, distinguishing between cases when the chain is connected by both ends to the same cluster (i.e., a loop), or a chain (unstretched or stretched) bridges two different clusters.

1. The first class are loops of type 1 (*r*_*e*_ ≤ 0.42 nm, mostly *r*_*e*_ ≈ 0.35 nm). Both ends of the chain are connected to groups within the same cluster. The groups themselves are nearest neighbors.
2. The second class is a loop of type 2 (0.42 ≤ *r*_*e*_ ≤ 0.65 nm). Similar to the previous type, loop 2 connects the interacting groups inside a single cluster. The groups, however, are not nearest neighbors.
3. The third class is that of unstretched chains (0.65 ≤ *r*_*e*_ ≤ 0.85 nm), that span two neighboring physical clusters. Such chains are flexible and the segments can move perpendicular to the chain backbone. They can reach relatively large distances away from the connecting line of the chain ends.
4. The fourth class is a stretched chain (*r*_*e*_ ≥ 0.85 nm), connecting two physical clusters. The chain segments reside on the connecting line between interacting groups.

As stated above, a SFC of certain class can be transformed into another class by deformation. The transformations are observed during the stretching of the sample. When the initial structure is simulated without deformation, however, the classes of SFCs remain stable.

We studied the transformation of the SFCs during the deformation. One might claim that they largely preserve the stationary state whereby all SFCs which are transformed into a different class are replaced by chains from another class that are transformed back into the particular class. For example, during the slow deformation 120 loops out of 640 were transformed into a stretched state, and another 120 stretched chains were transformed back into loops, indicating, that the total concentration of structural components did not change. These transformations are similar for slow and quick deformation.

**Figure 6:**
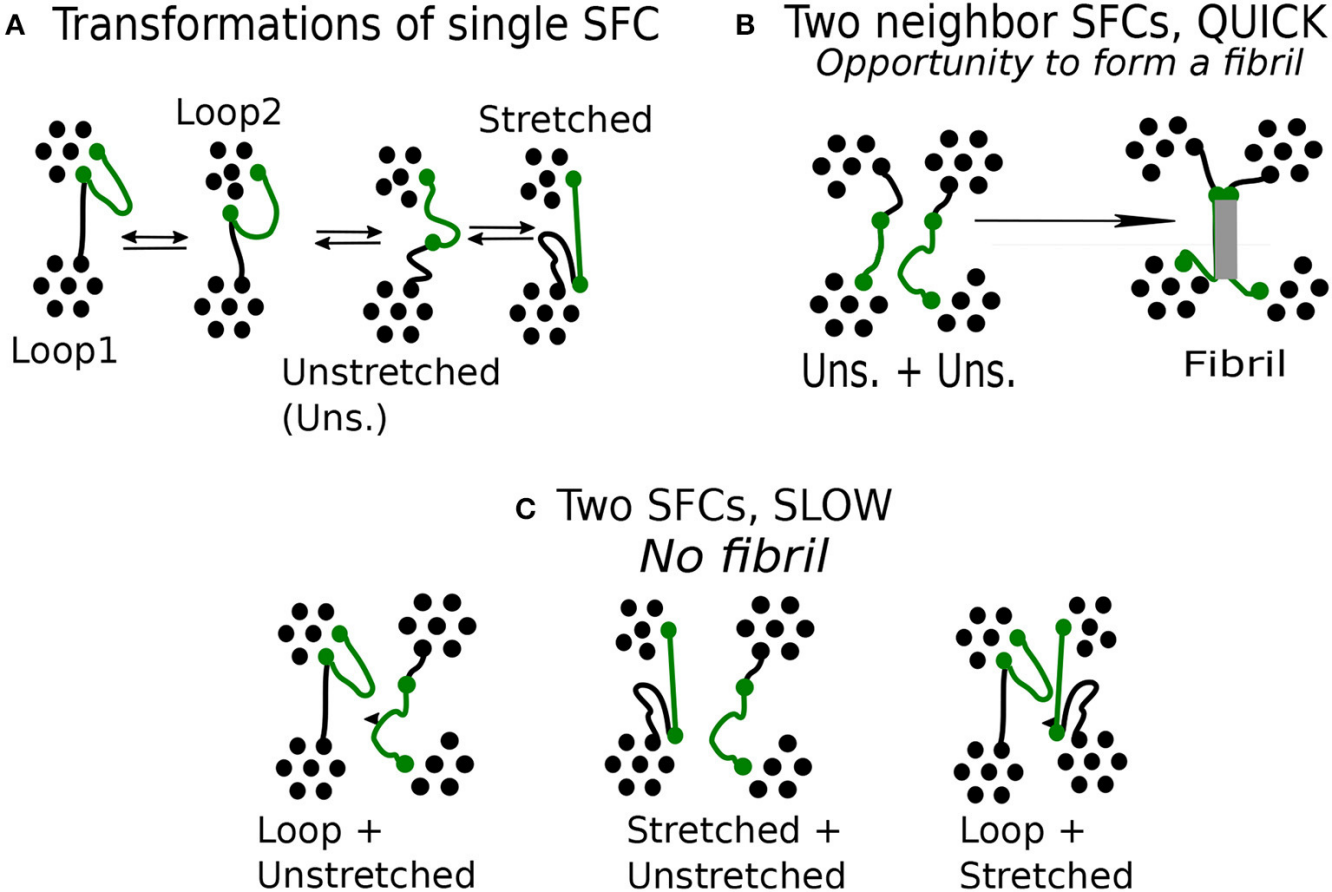
Mechanism of fibrillar structure formation in the network model: **(A)** Reversible transitions of single flexible chain (green). **(B)** Specific configuration of two unstretched chain, observed during quick deformation. **(C)** Specific configuration of two chains, observed during slow deformation.

The next specific property is that the transformations occur always between neighbor classes in a sequence of loop 1↔ loop 2 ↔ unstretched chain ↔ stretched chain, whereas transformations between extremes, loop 1 ↔ stretched chain are practically absent. Transitions that skip an intermediate class are seldom too. The observed transitions are reversible (see Figure 6A).

In the case of unstretched state, a chain has chance to form a fibril. To this end it is important to have more such neighbor chains in its vicinity. We find that a fibril is created when at least two chains start from an unstretched state (Figure 6B), then the respective transition turns irreversible.

We find that a favorable situation for the formation of fibrils emerges during quick deformation and this raises the question why the formation mechanism favors quick deformation and not a slow one.

The unstretched SFC remains unstable during a limited time. After that, it is transformed either to a stretched chain or a loop. Transition from a loop to a stretched state through unstretched state is induced by deformation. During a quick deformation, the chains change state nearly simultaneously as they have to comply quickly with the imposed stretching. Whenever this occurs, a fibril is formed. We find that a fibril is created when at least two chains start from an unstretched state (Figure 6B), then the respective transition turns irreversible.

In a slow deformation, the stretching of this chain takes 10 times longer than during the quick one. The neighboring chains may remain unchanged so that a given chain terminates its transformation before the neighboring chains begin to deform. The chain transformations are not synchronized and various combinations of neighboring chains can be observed (Figure 6C).

The formation of fibrils requires synchronous deformation of macromolecules. Favorable combination of SFCs, happens when both neighboring chains are in the class of unstretched chains. A fiber structure can hardly emerge from two chains which are in significantly different classes, say, one of them being a loop, or if one of the chains is stretched and the other—unstretched. The fiber formation is also unlikely for a pair of stretched chains.

### 3.2. The Role of Collective Alignment of Chains

Deformation changes the network topology. Ejection from the spider body in natural systems, or tensile deformation in our model, contribute to chains' alignment and ensure the necessary narrow orientation whereas the undeformed sample has random orientation of chains.

**Figure 7:**
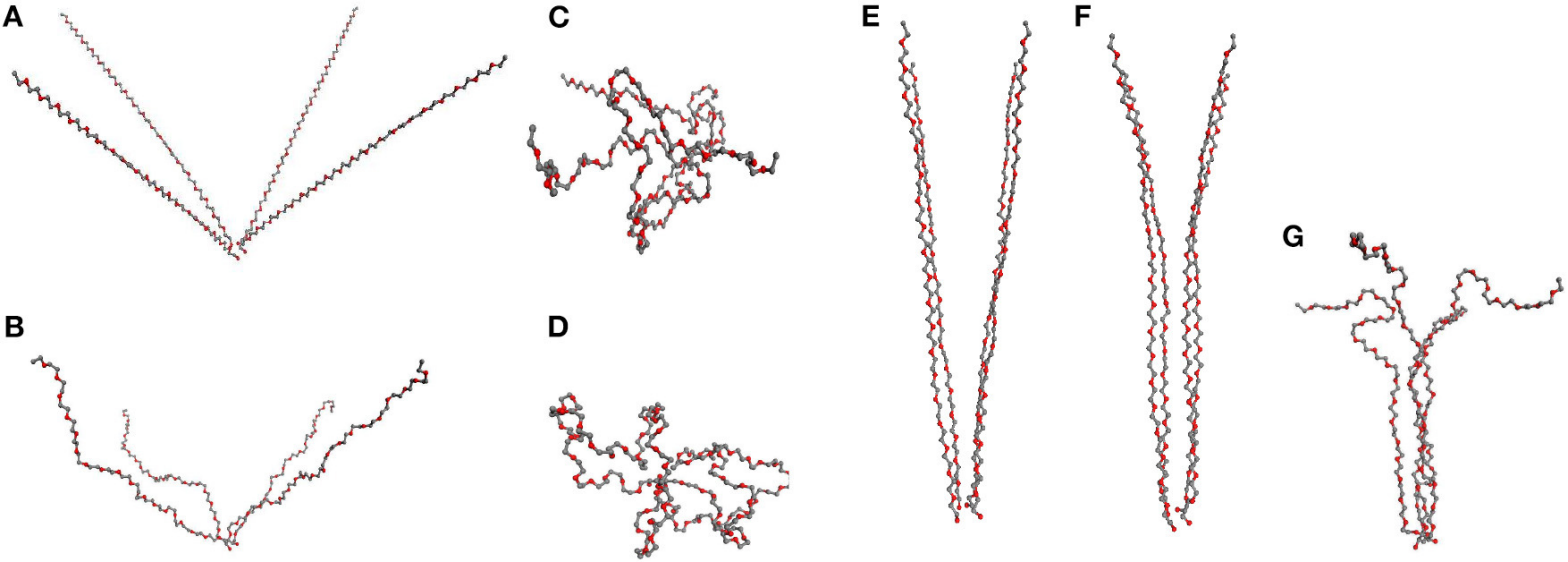
“Four chains model”: **(A–D)** 4 PEG chains initially at angle 50° with subsequent relaxation: **(A)** stretched chains, **(B)** unstretched chains; **(C)** loops of type 2, **(D)** loops type of 1. **(E–G)** PEG chains initially at angle 10°, relaxing subsequently to partially aligned stretched chains, **(F)**, in **(G)** a fibril after relaxation.

We find that for the formation of fibrils in the network model one needs a synchronous transformation of several SFCs residing initially in an unstretched state. Within the general network model the study of this behavior is complicated as there are too few neighboring chains with common orientation within the limited size of the sample and therefore they seldom deform simultaneously. The simultaneous deformation of several SCFs is readily examined within our simple model of four chains, each of which is here made of 60 segments, attached to a single physical cross-link while the opposite chain ends are fixed in certain distance from the physical cluster (Figure 7A).

We consider first the orientation of chains as it explains why fiber formation is not observed at small deformation of the network. In that case, the individual chains are mostly oriented at large angle (Figure 7A) regarding each other. The simulation starts with all the chains sufficiently stretched, then upon subsequent reduction of the end-to-end distances of the chains one observes two scenarios depending on their mutual orientation.

If the chains are not sufficiently parallel, they are transformed into loops and are not part of any organized structure (Figures 7A–D). The formation of fibrils in network models is observed only when the chains form a small angle (≤ 30°) as in Figure 7E, where the chains are mutually aligned at 10°. If the chains are gradually released and the end-to-end distance decreased, one finds as a result that the chains remain aligned (Figure 7F), with a subsequent formation of fibers in Figure 7G maintaining a stable fibrillar structure.

In our computer experiment we stop the chain relaxation in the moment when further compression of the chains requires energy and the relaxation is no more spontaneous. Even after that moment during the subsequent long simulation the fiber persists and its ordering appears to increase with time. The resulting structure can be broken only by an increase of temperature.

Consider now the impact of quick and slow deformation. Figure 7 describes the situation, when all chains are relaxed simultaneously, following a quick deformation of the network model (cf. Figure 6B). The chains, making originally a mutual angle ≤ 30° among themselves, form an unstretched fibril which remains relatively stable when compressed back to a rather small end-to-end distance of the chains.

A different situation is observed after slow deformation (see figure in Supplementary Material) when the relaxation of chains proceeds one by one. In our simulation we see no fibril created in that case. Instead, the stretched chains transform into loops.

**Figure 8:**
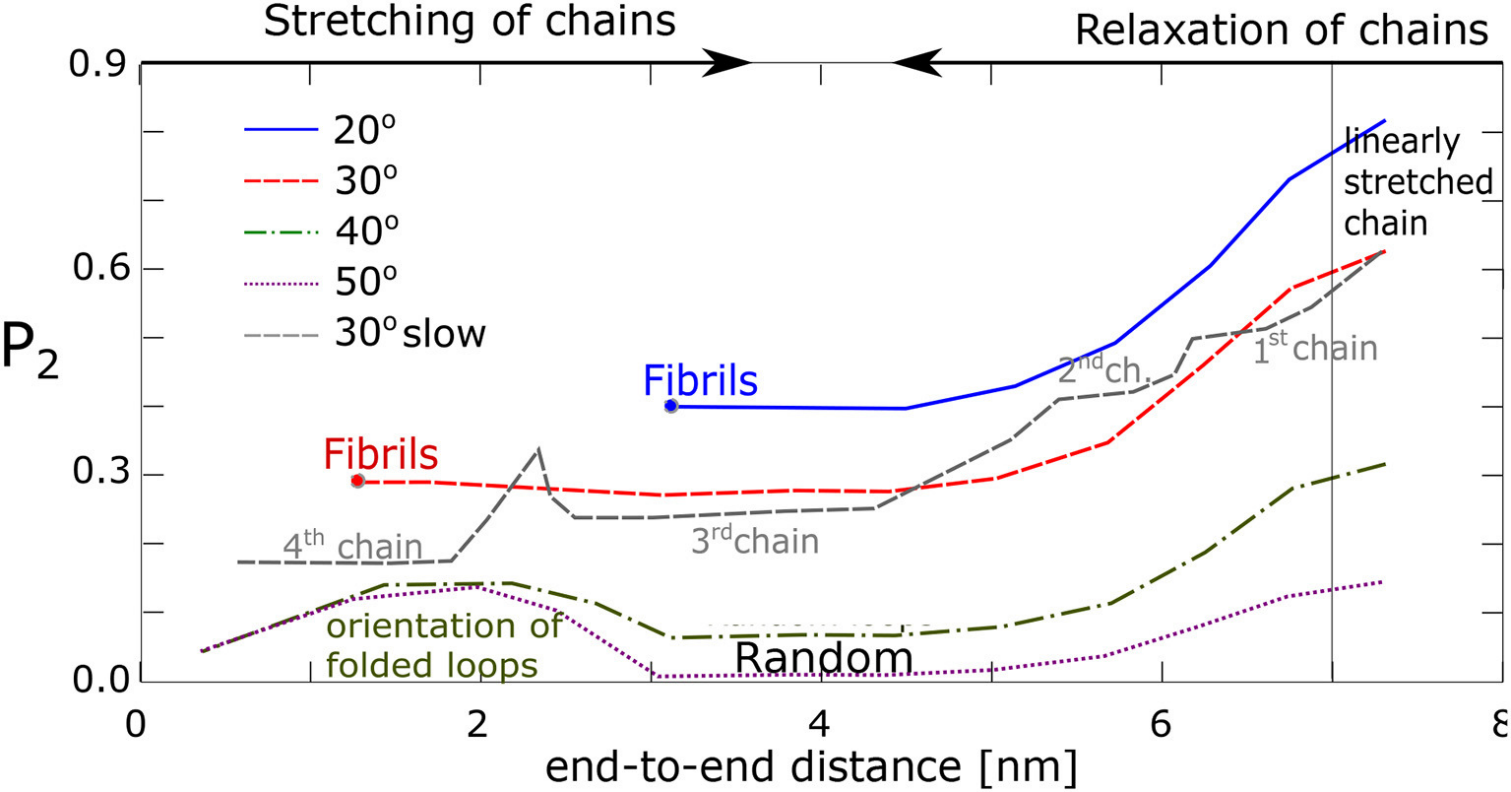
Alignment of chain segments with the fibril axis: An orientational order parameter, *P*_2_, as function of the chains' end-to-end distance during relaxation.

We have tried to quantify the process of fibril formation (assumed to orient along the *z*-axis in the simulation box) by using an orientational order parameter, *P*_2_(cosθ), Equation (2), where θ stands for the angle between the chain segments and the fiber vector, ${\vec{v}}_{F}$. For example, with each chain comprising 60 segments, θ is calculated from the vectors (${\vec{v}}_{1}=A_{3}-A_{1}$, ${\vec{v}}_{2}=A_{4}-A_{2}$, …, ${\vec{v}}_{58}=A_{60}-A_{58}$), and ${\vec{v}}_{F}$. Results are shown in Figure 8. In case of alignment, *P*_2_ stays positive, *P*_2_ ≥ 0.3, yet less than unity on average, indicating that the segments in fibrils are not perfectly oriented along the fiber vector. Apparently, for the chains, which make an angle 40° and larger (with no formation of fibrils), the value *P*_2_ becomes close to zero, which reflects a random orientation of the segment. Interestingly, even the chains transformed into random loops show some increased orientation in the end phase of simulation when the end-to-end distance becomes very small (≈2 nm), presumably as a consequence of a secondary orientation of loops.

The data from our four chains model agree with our previous findings from the network model. It was found that the chains with favorable geometry and interaction tend to form fibrillar structures with each fiber consisting of 2–8 chains attached to the same physical cross-link. To this end the stretched chains have to make an angle with one another, which is 30° or less. A second condition is a high enough stretching ratio. All chains must be present simultaneously in an unstretched state at a time. Fibril formation fails, when one chain is linearly stretched, or forms a loop. In linearly stretched chains the atomic groups from two macromolecules are usually too far apart and do not interact. On the other hand, when the chains reside as loops, the segments are disordered and alignment fails too.

### 3.3. Bundles of Macromolecules

In the previous sections, our basic structural unit was the short flexible chain (SFC). Several SFCs could be aligned under certain conditions into a stable fibril. It seems, however, that the route from several SFCs to fibril is not direct. There occur transient objects, which we refer to as bundles. Bundles are composed of 3 or more aligned SFCs, and they appear to serve as precursors to permanent fibrils. In contrast to fibrils, however, bundles can exist temporarily. In this section, we focus on the behavior of bundles. The SFCs, which form a bundle, can be either of stretched type or unstretched. As a rule, bundles connect two clusters of *AA*-groups.

One can analyze several aspects of bundle properties, for example their number and weight, change of orientation in space, rotation, and twisting. The basic analysis includes the number of the bundles per simulation box as well as the number of chains in the bundle. Average values and standard deviations were calculated in four independent samples of the network. We identified 7 ± 1 bundles during 10 × “quick” deformation, 9 ± 2 bundles during “quick” deformation of the box, and 10±2 in the slow deformation, so that the difference between slow and quick deformation appears statistically insignificant. The number of bundles looks small, yet, 22% of all atomic groups of the organic network participate in bundles. The bundles are mostly composed of 3 SFCs whereas bundles composed of four or five chains, occur rarely (on average, 0.625 bundles/box of four-chains, and 0.25 bundles/box of five-chains were detected).

Bundles of SFCs do rarely exist in the beginning of the simulation, and most of them appear during the deformation. The majority of bundles seems to appear in the final phase of the simulation, suggesting a predominantly dynamic nature of the process. The formation of bundles requires apparently certain time, whereby the segmental displacements of *AA*-groups play a role.

The behavior of bundles during simulation was observed. The average size of bundles is constant during the simulation, however, individual chains can fluctuate from one to another bundle. Regarding the geometry of bundles, we first examine the aspects, which are related to the formation of fibrils: rotation and twisting. These aspects were selected since they have different course in the slow and quick mode.

**Figure 9:**
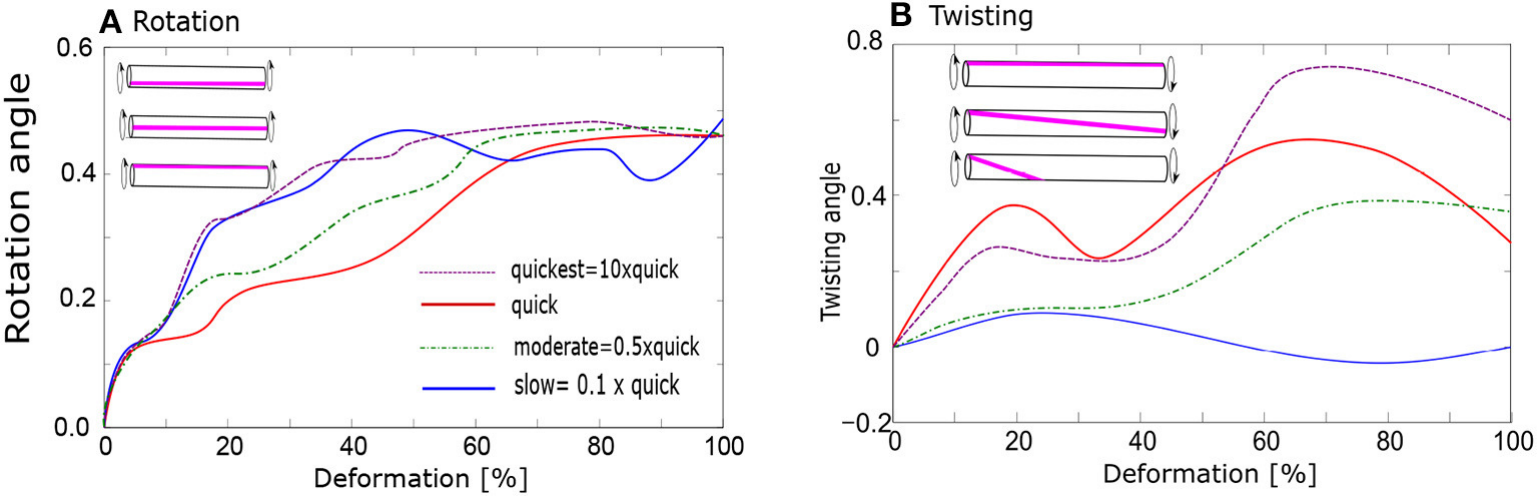
Evolution of bundle geometry during deformation; **(A)** Rotation of bundles: average rotation angle of bundles (in radians) from the beginning of the deformation; **(B)** Twisting of bundles: average twisting angle of bundles (in radians). Various stretching rates: 10× quick, quick, moderate, and slow.

In what follows we describe the rotation of the bundle about a rotation axis pointing along the bundle. One end of the bundle is at the center of mass of the chain ends, associated with the bundle, and the same applies to the other end of the bundle. Bundle rotation is then observed when the chain end-groups move with respect to bundle axis (see Figure 9A), whereby such rotation sets in from the very beginning of the deformation. During rotation both ends of the bundle may rotate clockwise or counterclockwise with the same rate, otherwise the bundle is twisted. The degree of twisting is given by the change of angle between two rotation vectors (Figure 9B).

We find significant difference in the average twisting intensity between slow and quick deformation. The twisting is enhanced during the quick deformation, where we observe also increased formation of stable fibrils (Figure 9B). Thereby all the deformation rates between 0.2× and 10× faster than “quick” deformations have similar effect. The twisting intensity also increases with growing degree of deformation.

No fibrils or bundles persist after the “slow” deformation, whereas almost 100% of fibrils persist after the “quick” deformation. The consequences of moderate deformation for the relaxation behavior are generally indicated in Figure 4c. Even if part of the fibrils is destroyed over the whole period of relaxation, 60% of them still persist after the onset of relaxation following a delay period, as in Figure 4c. These 60% participate in the twisting process.

We investigated the effect of twisting also in our four-chain model, starting with unstretched chains as an initial configuration. Upon twisting, the potential energy of the four chains was found to decrease whereas when this model was simulated without twisting, the energy remained constant. It was found that the chains can twist only slightly. The most stable atomistic configuration has a twisting angle 2.5°.

The model is simplified and therefore there are doubts about the validity of results mainly in relation to behavior of real hyrogels. From the structural aspects, our model resembles a micellar network with aggregates comprising cores, formed by hydrophobic Acrylic Acid (AA)-clusters, intermediate layer from poly-lactide-glycolide and shells, formed by hydrophilic polyethylene-glycol (PEG) chains. Model micelles are composed of two layers: core from AA, and hydrophilic PEG shell.

The proposed model provides insight into the relationship between gel structure and deformation response. The model response shows effects, which are observed in real hydrogel materials during deformation: hysteresis during forward- reverse deformation, yield stress, solvent redistribution during deformation (Zidek et al., 2017a). On the other hand, when we compare the model data to the experiment, the model values of stress appear significantly higher than the experimental ones. The limitations of mechanical response are discussed elsewhere (Zidek et al., 2016).

The model structural changes accompanying the fiber formation are minimalistic and we cannot find a corresponding experimental study of of real hydrogels on the atomistic level. However, the model deformation response matches that observed in real materials. As well the final structure is similar to the structure of materials with bundles and fibrils. That allows us to assume that the model evolution of network is similar to the evolution of real materials.

### 3.4. The Behavior After Deformation: Network Relaxation

One can compare the relationship between time of deformation and relaxation. While the deformation is controlled by the user and it depends on stretching rate and stretching ratio, the relaxation is a spontaneous process, which cannot be influenced by the operator.

The relaxation was analyzed by means of time-autocorrelation functions which were fitted by exponential decay. A characteristic parameter of the exponential function is the half-time of relaxation. Our network reveals several relaxation processes with significantly different relaxation times:

- Inertial motion of the atoms is calculated from the velocity autocorrelation function. This function describes the inertia of atomic groups. It was computed within 1 ps whereby the successive atomistic configuration was recorded every 1 fs.
- Relaxation of bonds and bond angles. It was calculated from the autocorrelation function of the covalent bonds over 100 ps, whereby the configurations were recorded every 0.1 ps.
- Relaxation of conformations involves rotation of bonds and change of conformations. As with the previous item, we applied the autocorrelation function of the covalent bonds within the same time range.
- Segmental hops in physical clusters. The physical clusters are cores of micelles in our model. During the segmental hops, the concentration of physical clusters in the box does not change while the interacting groups only switch from one cluster to another (performing segmental hops). The autocorrelation function identifies whether the interacting *AA* groups are still present in the initial physical clusters. The time interval of relaxation simulation is 100 ns and configurations are recorded each 0.1 ns.
- Bundles or fibrils (in the relaxation analysis they are not distinguished) change their axis orientation in space. The time autocorrelation function of bundle orientation was calculated from a 100 ns period with interval of 0.1 ns.

For comparison with the derived relaxation times, we recall that the quick deformation takes 10 ns, and the slow one lasts 100 ns, as the most relaxation processes are significantly faster than the deformation. Only the relaxation of physical clusters and bundles appears comparable to the interval of deformation.

The relaxation times were calculated for several stretching rates. The fastest stretching rate is 10 × faster than the “quick” deformation considered in the previous section. The range between fastest and slow deformation was divided into seven intervals on a logarithmic scale. A table of all relaxation times is presented in the Supplementary Material. Here we analyze the relaxation times in terms of relaxation after quick and slow deformation as in previous section.

**Figure 10:**
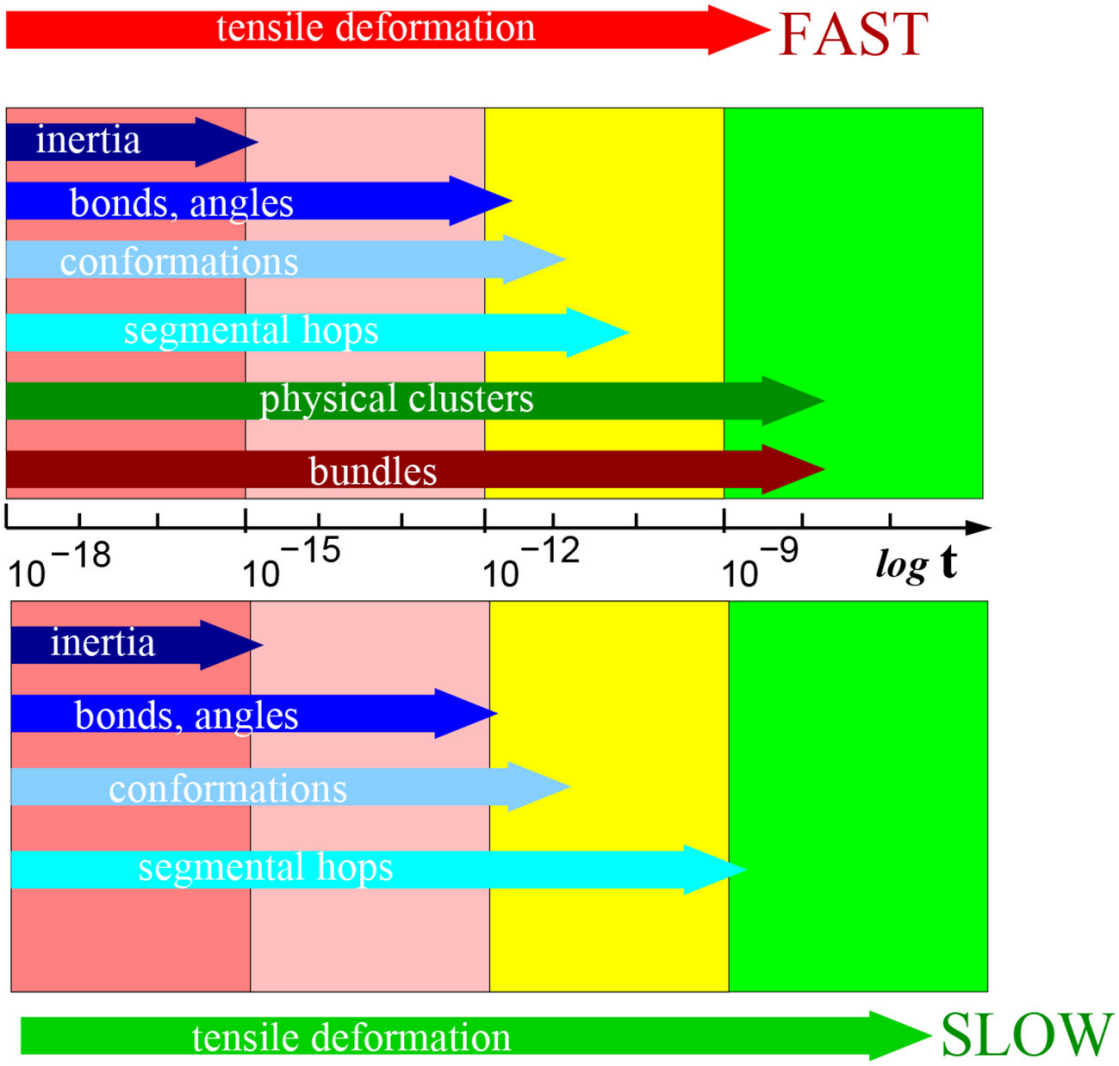
Schematic presentation of relaxations times in [s] after slow (below) and fast (above) deformation process (on a logarithmic scale). Particular structural elements are indicated in the horizontal arrows marking the typical time interval length. A table of all relaxation times is presented in Supplementary Material.

A summary of the typical relaxation times after the deformation is presented in Figure 10. Several femtoseconds (fs) after the deformation is the interval, where the motion of atomic groups is driven by inertia. One can recognize other relaxation processes at times significantly longer than the inertial motion. The loss of inertia, driven by thermal motion, was observed also in the reference sample without deformation.

The next process pertains to the fluctuation of bonds and angles. The relaxation process is observed within the range of hundreds of fs. It describes the reorientation of covalent bonds. The relaxation is driven by thermal motion of molecules as it was observed also in the reference sample without deformation.

The conformation relaxation by rotational motion of chain segments occurs within tens of picoseconds. This motion is predominantly driven also by thermal motion, however, the degree of deformation plays a role too.

The process of segmental displacements is related to the reconstruction of micellar structure and takes place within the range of nanoseconds. Macroscopically, the micellar structure does not change, but the individual physically interacting atomic group can switch from one to another physical cluster (core of micelle). That relaxation process is unambiguously a response to deformation. We did not observe it in the reference simulation box without deformation.

During the longest interval of tens of nanoseconds one observes a reorientation of bundle axes. The reorientation implies rotation of the bundle. In the same time, we observe also a second wave of segmental hops as a means of reconstruction of the physical clusters. We can assume that in that interval, the bundles are stabilized and this affects the physical clusters.

We observed a relaxation of bundles at the intermediate stretching rate (0.5× quick) along with temporarily persistent fibers. On the other hand, at the stretching rate 0.2× “quick” the relaxation of bundle orientation cannot be analyzed, because bundles are not stable. As well the recovery of physical network starts immediately after deformation.

Bundle axis orientation and the accompanying segmental hops are specific to the fast deformation. They are not observed after the slow simulations or without deformation.

**Figure 11:**
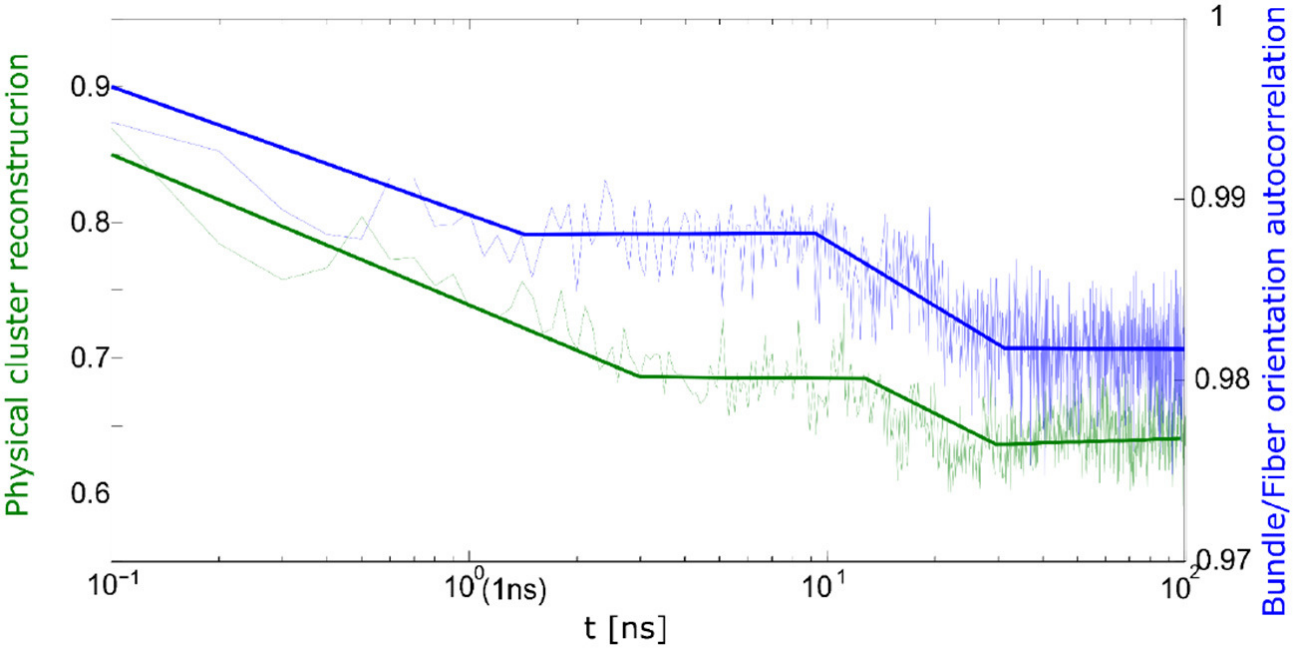
Correlation between bundle orientation (blue) and cluster reconstruction (green) during relaxation process.

The correlation between cluster reconstruction and bundle orientation is presented in Figure 11. The bundle orientation autocorrelation function is compared to cluster reconstruction autocorrelation. From Figure 11 it appears that the two processes take place simultaneously.

Thus, during the relaxation one can observe the differences between quick and slow deformation. During the slow deformation predominantly the physical network undergoes relaxation whereas after the quick deformation one observes a relaxation of the bundles and fibrils.

The relaxation times can be used to determine the Weissenberg number (*W*_*i*_) as a dimensionless quantity, which indicates the typical ratio of elastic and viscous forces in our structure. *W*_*i*_ is calculated from the deformation rate ($\dot{\gamma }$) and the respective relaxation time τ of the particular process.

(4) $$\begin{array}{l} W_{i}=\frac{F\left(elastic\right)}{F\left(viscous\right)}=\dot{\gamma }\tau \end{array}$$

The *W*_*i*_ was calculated for each relaxation process and each stretching rate. All numerical values are presented in the Supplementary Material.

Table 2 shows the values of deformation rates, relaxation times and Weissenberg numbers (*W*_*i*_) for selected deformations and relaxation processes. The processes can be subdivided into 3 groups: viscous (v) (*W*_*i*_ < < 1), viscoelastic (ve) (0.5 < *W*_*i*_ < 2), and elastic (e) (*W*_*i*_ >> 1).

**Table 2:** Relaxation times (t_*r*_*el*) and Weissenberg numbers (*W*_*i*_) for various relaxation processes at different stretching rates; viscous (v); elastic (e); viscoelastic process (ve).

**Deformation rate [ps^−1^]**	**Conformations t_*rel*_[ps]/ *W*_*i*_**	**Segmental hops t_*rel*_[ps]/ *W*_*i*_**	**Bundle t_*rel*_[ps]/ *W*_*i*_**	**Fibril formation**
10 × quick 10^−3^	21.3/0.0213(v)	1,776/1.773(ve)	57,600/57.6(e)	Yes
Quick 10^−4^	19.0/0.0019(v)	760/0.076(v)	20,300/2.03(ve)	Yes
Slow 10^−5^	10.0/0.0001(v)	2,100/0.021(v)	–	No

The Weissenberg number clarifies the correlation between the relaxation times and formation of fibers. The simulations, when we observe complete recovery of the structure, contain only the viscous processes according to *W*_*i*_. The other simulations, when we observe the formation of fibers, show either the viscoelastic reorientation of molecular bundles or viscoelastic reconstruction of physical clusters or micelles.

### 3.5. Unidirectionally Alternating Structure of the Fibrillar Phase

It has been mentioned in the Introduction that the fibrillar structure of self-assembled natural materials reveals an alternating sequence of stiff and soft blocks of proteins (quasi-crystalline blocks of β-sheets and amorphous layers rich in poly-glicine) (Nilebäck et al., 2018).

**Figure 12:**
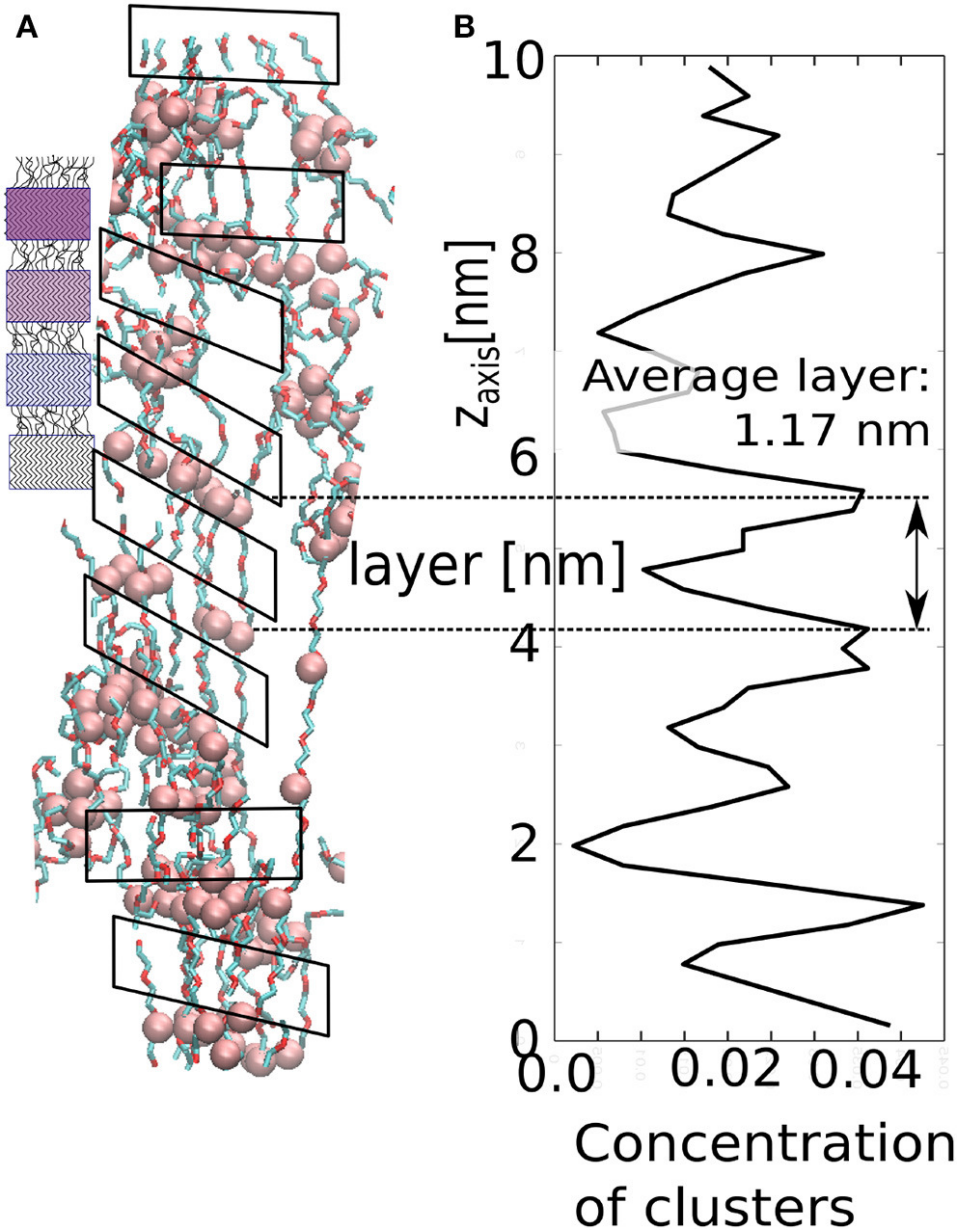
Structure of the stretched network in our model: **(A)** small figure—schematic picture of spider silk; large figure—simulation snapshot; **(B)** local concentration of AA-clusters as along the z-axis.

Our model demonstrates similar structural evolution to fibrillar phase upon strong and fast deformation in z-direction. An alternating sequence of layers of micelles and layers of stretched chains (solated fibrils) is also observed in the deformed sample as demonstrated in Figure 12. The layers enable the material to hold constant span between the aggregated layers of clusters. In our case, the stretched chains are rigid segments, whereas the cluster with loop chains have viscoelastic behavior. The layer arrangement can be derived from the local concentration profile along the z-axis. Although the periodic arrangement is not perfect, one can detect an average distance between adjacent maxima. The average span is ≈1.17 nm, which is the length of stretched chains with a layer of AA-clusters.

## 4. Conclusions

In this work we present molecular dynamics simulations of a model, which shows the ability to form ordered fibrillar structures on molecular level. A significant result of our study is the finding that the relevant mechanism is sensitive to strain amount and rate. The fibrillar structure is formed only by both quick and large deformation.

We identified some factors, which are important for the formation of nanostructured fibrilar material. During deformation, one must achieve synchronized unfolding of macromolecules during deformation. The synchronized unfolding is activated by quick deformation. Another aspect is the ability of the chains to form bundles of aligned macromolecules. Such bundles can be stabilized by twisting and thus transformed into fibrils. Whether the macromolecules can form fibrils depends on the molecular structure. The molecular structure has been investigated here by a simplified four-chains model, which elucidates the conditions necessary for a material of certain composition to form fibrils.

The structural transformations are reflected in the relaxation process of the sample. The relaxation after fibril formation is manifested in the reorientation of fibrils and bundles. In the case of slow deformation, where fibrils are not formed, the process of reorientation is not observed.

The present model makes it possible to consider new materials, which are able to form fibrillar structure. The primary structure according to our findings can be produced in a laboratory. When one combines an appropriate primary structure and satisfies the conditions of preparation/extrusion, a nanostructured fibrilar material similar to spider silk can be achieved also in a laboratory.

## Data Availability Statement

All datasets generated for this study are available within the article and from the corresponding author on request.

## Author Contributions

JZ carried out the MD calculations and analyzed the data. JZ, AM, and JJ interpreted the results and developed the theoretical scheme. JZ and AM wrote the paper.

### Conflict of Interest

JJ was employed by the company SCITEG a.s. The remaining authors declare that the research was conducted in the absence of any commercial or financial relationships that could be construed as a potential conflict of interest.

## Supplementary Material

Section 1 Setup and parameters of molecular dynamics simulations.

Section 2 Description of the four-chain model.

Section 3 Description of model objects.

Section 4 Data from relaxation of hydrogels.
