# Supplementary material for: Dynamic Responsive Formation of Nanostructured Fibers in a Hydrogel Network: A Molecular Dynamics Study
Source: Front Chem. 2020 Feb 26;8:120. doi: 10.3389/fchem.2020.00120 (PMC7054485; doi:10.3389/fchem.2020.00120)
Supplement: Section 1 — Setup and parameters of molecular dynamics simulations. [file Data_Sheet_1.PDF]

Supplementary Material

1 NETWORK MODEL

The deformation of the sample was investigated by means of our network model. The system comprises a representative cubic box of hydrogel network with 5 nm side size. The model is composed of 8 macromolecular chains with each chain comprising 200 molecular groups. The macromolecular phase is solvated by 2447 molecules of water. The motion of atomic groups of chains and of the solvent was modeled by molecular dynamics simulation. A list of parameters is given in Table S1.

1.1 Interactions

The sequence alternates flexible poly(ethylene glycol) (PEG) chains and interacting groups, which are modeled as acrylic acid (AA)-groups. The AA-groups have prescribed strong intermolecular interaction of (50 kJ mol^{-1}). The chains are periodic and entangled, therefore, a chain cannot collapse into a globular state, maintaining thus the macromolecular network. Each polymer is modeled as a freely rotating chain (FRC) with constant length of the segments, bending angle between two successive segments, and a random angle of rotation. The atomic groups are connected to the chain by unbreakable strong bonding interaction. The amplitude (strength) of the bond

Table S1. List of parameters used in the simulations

Parameter	Value
Partial charge on oxygen in PEG chain (e - elementary charge unit)	-0.24 e
Partial charge on methylene in PEG chain	0.12e
Properties of simulation	
Number of PEG chains	8
Number of monomeric units in each chain: 1monomer = 3 beads($CH_2 - CH_2 - O$)	60
Number of interacting AA groups forming the physical crosslinks in each chain	20
pH	Acidic
Interaction energy between AA-groups at given pH	50 $kJ\ mol^{-1}$
Interaction energy PEG-PEG	0.34 $kJ\ mol^{-1}$
Interaction energy PEG-WATER	0.67 $kJ\ mol^{-1}$
Temperature	300K
Box size (in undeformed state)	5 nm
Particles present in the simulation box	
PEG- CH_2 atomic groups (partial charge dependent on PEG oxygen)	960
PEG- O atomic groups (partial charge variable)	480
PEG- interacting AA -Acrylic acid group (Partial charge 0)	160
WATER Hydrogen (partial charge 0.425 e)	4894
WATER Oxygen (partial charge -0.85 e)	2447
Molecular dynamics method	
Software GROMACS/ ensemble	NVT
Standard leap-frog md integrator	md
Thermostat	V-rescale
Electrostatic interaction - Particle-Mesh Ewald electrostatics.	PME
Type of deformation	Tensile
Stretching ratio	100%
Deformation rate quick	0.1 ns^{-1}
Deformation rate slow	0.01 ns^{-1}

harmonic potential is chosen as 3.3×10^5 kJ mol^{-1}, and the bond length is $0.153\,nm$. The large force constant of the harmonic bonding potential has a consequence that the bonds are virtually rigid with almost no fluctuation of their length. The next parameter of FRC, the bond angle, is 109.5^o, and the force constant of this angle is 418 kJ mol^{-1} so that the angle is not quite rigid. It can be deformed, when the local force is sufficiently strong, enabling thus the relaxation of the network. Since the chain is taken as freely rotating, a dihedral potential was not included. The electrostatic interactions were modeled by Particle Mesh Ewald algorithm (Essmann et al., 1995-11-15). The box is solvated according to the SPC-E simple point charge model of water (Miyamoto and Kollman, 1992). Water molecules fill the space between the macromolecules within the network. The energy was calculated from a contribution of various types of potential energy. The Hamiltonian of the simulation is:

$$\mathcal{H} = \sum_{nb} U_b + \sum_{na} U_a + \sum_{n} U_{el} + \sum_{n} U_{LJ} \tag{S1}$$

where the bond and angle potentials were calculated from the harmonic potential equations.

$$U_b = Kb\,(r - r_0)^2\,, U_a = Ka\,(a - a_0)^2\,, \tag{S2}$$

where constants Kb and Ka are force constants of the bond(angle) and r_0 and a_0 are are equilibrium bond length and angle.

$$U_{lj} = \frac{A}{r^6} - \frac{B}{r^{12}}. \tag{S3}$$

All pair Lenard Jones interactions have depth of energy between CH_2, O, AA were in range from 0.3-0.7 kj mol-1 (from GROMOS54a7 force field). The pair interaction AA-AA groups is $50kJmol^{-1}$. The electrostatic interation is calculated from the Coulomb equation:

$$U_{el} = \frac{1}{4\pi\epsilon_0}\frac{q_1 q_2}{r} \tag{S4}$$

The electrostatic force includes all charged groups, methylene ($q = 0.12e$) from PEG , oxy ($q = -0.24e$) from PEG, Oxygen and hydrogen from water have charges from SPC-E model. The model box has been stretched and the deformation response was monitored. The model has two variable parameters: the stretching rate and the stretching amount. The GROMACS software has a functionality of deformation acting simultaneously with the molecular dynamics simulation. In this study, the box was deformed at two stretching rates: slow $0.01ns^{-1}$, and quick $0.1ns^{-1}$. During the stretching along the z-axis, the box was compressed in xy-direction in order to conserve constant volume. The deformation of the system is reflected by the change in energy density, which can be transformed itself into a stress-strain relationship (cf. (Zidek et al., 2016, 2017)).

2 FOUR CHAINS MODEL

A disadvantage of the network model is that one cannot specifically control the mutual position of the chains, in particular, the combination of angles between stretched chains as well as their end-to-end distances, even though the network model shows the evolution of the entire network structure.

The 'Four Chains' model, in contrast, deals with four poly(ethylene glycol) chains, designed to describe the behavior of a small segment of the whole network. It provides full control of the mutual position of chains. All 4 chains have thereby well defined end-to-end-distance so that one can stretch all of them simultaneously, or alternatively, one by one.

A model parameter, reflecting chains' mutual orientation, is the angle between neighbor chains. It is a constant parameter, which does not vary during the simulation so that each set of simulations has a well defined mutual angle, which is kept constant. The mutual angle of the most strongly oriented chains is 5°, and the most disordered chains have orientation angle 50°. This model has been designed using Materials Studio (MS) software, Accelrys, Cambridge, UK, which provides control of the end-to-end distances while simultaneously the molecular dynamics simulation is carried out. The standard configuration described here is an initial structure of linearly stretched chains. Then the actual end-to-end distance is gradually changed with time while the MD simulation proceeds. The chains can be transformed into three configurations: stretched chains, loops (folded chains), and chains in some intermediate state. Eventually, the model structure evolves to either fibers or folded chains.

We characterized the resulting fibril shape by means of the 2nd Legendre Polynomial (P_2) which describes the orientation of different segments of the chains with respect to the fiber vector. The latter is the sum of end-to-end vectors of all four chains. P_2 describes the correlation of the fiber orientation with the individual segments of the chains, whereby $P_2 = -0.5$, when the segments are oriented perpendicular the to fiber, $P_2 = 0$, for randomly oriented segments, and $P_2 = 1$, when all segments are parallel to fiber:

$$P_2 = 0.5(3\langle\cos^2\theta\rangle - 1), \tag{S5}$$

where the θ is the angle of a segment with the fibril vector. An example of application of four chain model is presented in Figure S1. In it the relaxation of chains proceeds one by one (Figure S1a-e). It simulates relaxation during slow deformation whereby no fibril are created. The stretched chains in Figure S1a transform into loops Figure S1e. The same initial configuration of four stretched chains at angle 30°, however, relaxed simultaneously develops a fibril, Figure S1f.

3 COMPONENTS OF THE MODEL NETWORK

3.1 Short Flexible Chain (SFC)

The model is composed of macromolecular chains (of a given sequence), physical clusters of AA-groups, and micelles, whose shape and functions are similar to their counterparts in real materials. The basic structural components, which can be found in both models are short flexible chain (SFC) and fibrils. A short flexible chain (SFC) is part of a chain, which is bounded by two interacting neighboring AA-groups. It must be distinguished from the macromolecular chain. The PEG monomers of a given SFC move independently and are sensitive to nonbonding interactions from the surrounding network only. On the other hand, the motion of AA-endgroups is strictly limited as they are connected to some cluster and can only switch to another cluster. The four chains model is illustrated in Figure 3 in the paper. According to the end-to-end distance, one can specify the SFC as a loop, unstretched chain, or a stretched chain.

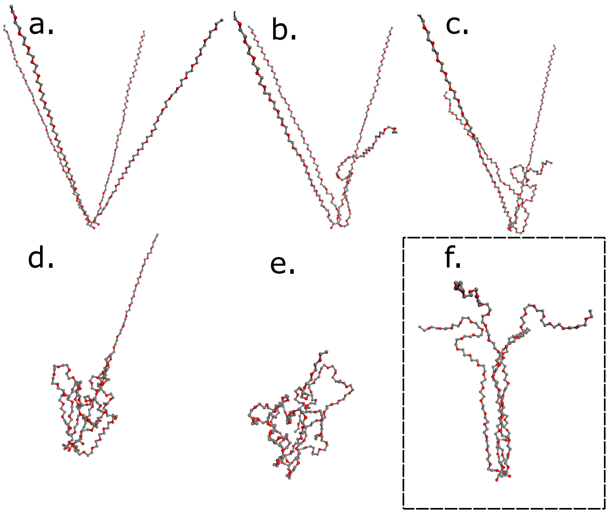

Figure S1. Four chains model with polyethylene glycol: chains at angle 30°, relaxing one-by-one: a) stretched chains, relaxation of the b) first-, c) second-, d) third-, and e) fourth chain; f) the same model in quick regime (all chains relaxed simultaneously).

The four chains model is composed of four SFCs. One end of each is fixed in exact position and the second end can be moved in direction of the end-to-end vector. The parameters are end-to-end distance and mutual angle of the SFCs.

3.2 Bundle

An object is identified as a bundle, when it satisfies three conditions:

- The bundle must be composed of 3 or more aligned short flexible chains (SFCs).
- The end-to-end distance of all SFCs in the bundle must be sufficiently long, $> 0.65nm$.
- The bundle connects only two physical clusters.

Bundles were detected in the simulation box during deformation and appeared as prerequisite to the fibrils. The bundles were identified in the highly stretched network and were analyzed in several aspects. Similar to fibrils, they are described by a the vector of the bundle, $\vec{v}$. The end point of a bundle, M_2, is the center of mass of end-groups of the chains, while M_1 is the starting point of the axis $\overrightarrow{M_1M_2}$. This vector characterizes the length and actual orientation of the bundle in space. It is calculated at every deformation ratio in the network. For a more detailed analysis one needs to define an exact orientation of the bundle. For each end-group we calculate the rotation vector $\vec{v}_{rot}$ of the end-group, which is perpendicular to the bundle axis. By the axis vector and rotation vector, we know the position of each end group in the bundle in every moment of the deformation. This is important for the analysis of bundle twisting. The bundles are defined also in the four chains model. They are identified, when the individual SFCs are aligned. As a rule, bundles connect two clusters of AA-groups. In this work we analyze several aspects of bundle properties, as indicated in Figure S2), and in particular, their number and weight, formation during deformation, change of orientation in space, rotation, and twisting.

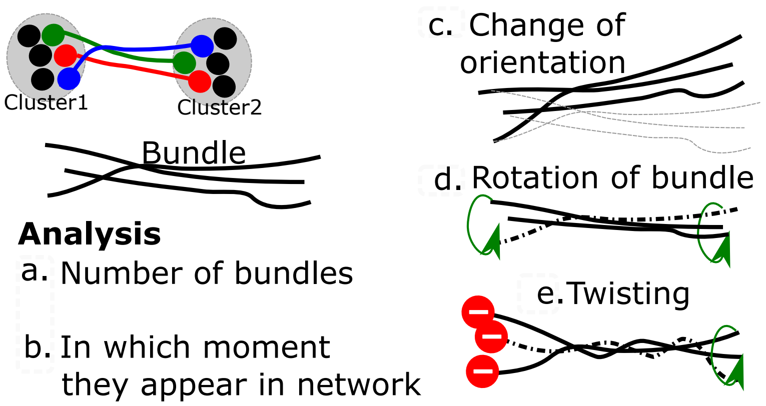

Figure S2. Bundle properties, $a. \div e.$, analyzed in this study.

The basic analysis includes the number of the bundles per simulation box as well as the number of chains in the bundle. Average values and standard deviations were calculated in four independent samples of the network.

3.3 Fibril

As mentioned above, the third element of the structure is the fibril. The fibril is formed from a bundle and it retains its shape after tension disappears in contrast to the bundle. We have verified that a bundle was transformed into a fibril in the network model. The fibril was detected by reverse deformation, when the fibrillar structure still persisted. This phenomenon was observed in the quickly stretched network with 100% deformation (Zidek et al., 2016). The fibril in the four chain models can be detected by removal of the constrains on chain ends. In that case, the fibril retains its shape, whereas the bundle in general may disintegrate.

3.4 Physical clusters

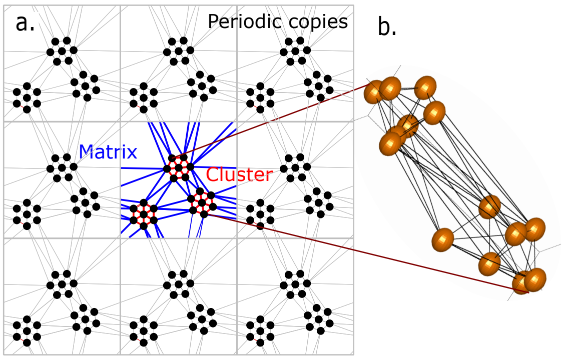

Figure S3. a) Delaunay triangulation of two selected AA-clusters in the simulation box (periodic images of the box are also shown). b) 2D-Scheme of triangulation. Orange spheres: interacting groups in clusters (cores of micelles), black lines: visualized results of Delaunay triangulation.

As a consequence of the deformation, a damage of the physical network may emerge. The damage affects the micellar structure of the network. One can examine the cores of micelles, which form physical clusters (PCLs) in the network. The strength of a physical network is proportional to the volume fraction of PCLs. Therefore, the damage can be estimated as a decrease of the volume

fraction of PCLs during recovery. The volume fraction is calculated in this work by triangulation of space between the interacting groups, namely, by $3D$-Delaunay triangulation (DT). DT is itself a mathematical algorithm, which creates the triangular mesh from the interacting AA-groups in 3-dimensional space. The main advantage of DT is that it can detect the set of nearest-neighbor interacting groups. The application of $3D$-Delaunay triangulation is readily available. The method is included in a Quickhull software library of algorithms (Barber et al., 1996). This library is included in MATLAB software, it can be linked to a $C++$ software code too. In the present study the DT was performed by selecting the coordinates of AA-groups in the simulation box and calculating the periodic images of the system, Figure S3a. Then, the $3D$-DT was performed as shown in Figure S3b. The result is a set of connecting lines, which span the nearest neighbors. This set of connection lines was then analyzed regarding the length of lines. In addition, it was determined what part is present in the central box when the line crosses the boundary of box. Classifying all lines according to their length, it is assumed that when a line is shorter than $0.4nm$, it is considered inside the cluster whereas longer lines are outside the cluster. The relative fraction of shorter lines (with respect to their length) vs. that of all lines in the box gives the volume fraction of clusters.

4 STRUCTURAL RELAXATION

The relaxation follows immediately after the deformation. For the relaxations, we prepared the set of deformation simulations at seven stretching rates. The sample was deformed to 100% stretching ratio in 1, 2, 5, 10, 20, 50, and 100 ns. The set includes two deformation rates from the article: quick (100% in 10 ns) and slow deformation (in 100 ns). Each deformation rate was followed by three relaxation simulations: 1 ps, 100 ps, and 100 ns. The relaxation simulations started immediately after deformation and the simulation time was divided into 1000 intervals, where the atomistic configurations were exported. Each relaxation simulation has its characteristic property, which was analyzed

4.1 1 ps simulation

The shortest 1 ps simulation was analyzed by of velocity autocorrelation function:

$$f(t) = \langle \mathbf{v(0)} \cdot \mathbf{v(t)} \rangle \tag{S6}$$

where v(0) is normalized velocity at time zero and v(t) is a velocity of atomic group at time t. The velocity autocorrelation function indicates the inertia of the atoms in the model.

4.2 100 ps simulation

The bonds orientation autocorrelation is calculated from the reorientation of vectors of covalent bonds in the dry phase. The bond-vector autocorrelation function detects the stability of the soft chains. The function value 0 means reorganization of soft network phase. It is calculated:

$$f(t) = \langle \mathbf{b(0)} \cdot \mathbf{b(t)} \rangle \tag{S7}$$

where b is a normalized vector of covalent bonds at time 0 and at time t. We found that in short time the bonds oscillate weakly and the bond angles fluctuate noticeably. At longer times one observes rotation of bonds and change of conformations. The half time of the process is calculated

from the exponentially decaying function

$$f\left(t\right) = \exp\left(-\frac{t}{\tau}\right) + C \tag{S8}$$

where τ is a time constant and the halftime of the process is calculated as $\tau ln2$. The C is a constant. The bond orientation autocorrelation function can be fitted by two term exponential decay functions (Figure S4a), suggesting the existence of two independent processes. The exponential decay means a first order kinetic process, which starts from the beginning of simulation.

4.3 100 ns simulation

In this time inerval, we calculated the correlation of interacting groups in the physical clusters. At each step, the clusters are identified by cluster analysis. By this method we detect the distribution of interacting groups within the individual clusters. Thus, all pair combinations of interacting AA groups in the same clusters were further analyzed in the next steps.

$$f\left(t\right) = \left\langle p(0) \cdot p(t) \right\rangle, \tag{S9}$$

where p(t) indicates presence (value 1) or absence (value 0) of the pair interacting groups at a given step. The average value from all pairs in the simulation box is continuous function in interval $\langle 0, 1 \rangle$, where 1 means, that the clusters are stable and 0 means, that the clusters were completely reorganized. The value between 0 and 1 means that the clusters were partially reorganized. The correlation function was fitted by combination of exponential decay and Boltzmann sigmoidal function (Figure S4b). The sigmoidal function is defined as

$$f\left(t\right) = 1 - \frac{C}{1 + \exp(\frac{t_0 - t}{slope})}, \tag{S10}$$

where C is a constant. The exponential decay means a first order kinetics as in the previous section. The Boltzmann sigmoidal function means that the process started with some delay after the beginning of simulation. The half-time is given by the inflexion point of sigmoid (t_0).

Eventually, a bundle (fiber) reorientation was studied. In that phase we do not distinguish between bundle and fibers. The correlation function is calculated from the orientation vector of the bundle axis. The fuction was calculated from small number of bundles. That is why it fluctuates significantly and it was not fitted by any function. The times were only estimated.

4.4 Relaxation times

The half times from all relaxations were collected in Table 2 and compared to the time of deformation. There is no change in the velocity autocorrelation functions, which was almost identical for all stretching rates. The relaxation of bonds and conformations is similar for all stretching rates, however, the quick stretching rate leads to slightly higher relaxation times. Cluster reconstruction and bundle reorientation are observed solely in the quick deformation.

4.5 Weissenberg number

The Weissenberg number(W_i) is calculated from the relaxation times. W_i can be interpreted as a ratio between elastic and viscous forces and is calculated from relaxation time (τ) and the

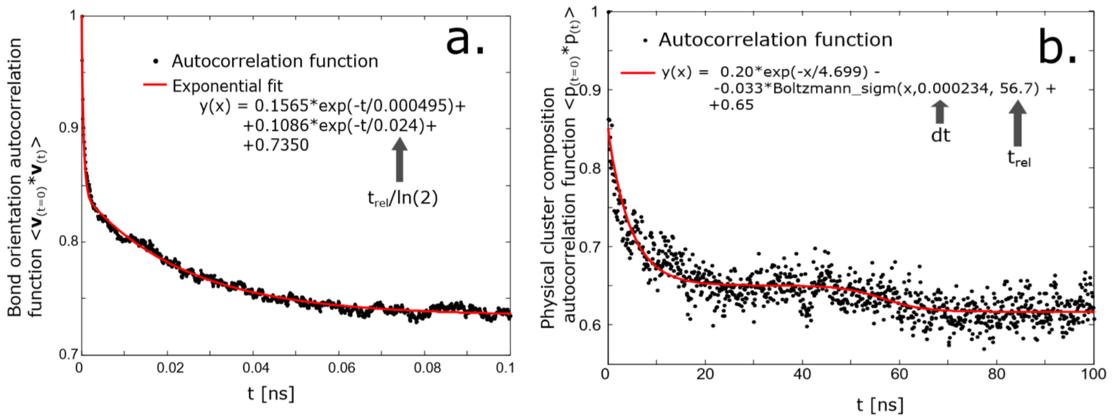

Figure S4. Fitting of autocorrelation functions a) bond orientation autocorrelation functions fitted by two terms exponential decay, and b. cluster reconstruction autocorrelation function-combination of exponential decay and Boltzmann sigmoidal.

Table S2. Times of deformation and relaxation [ps], The deformation time was set by the operator, the relaxation is a natural response of the material, N.D.= not detected

Time of deformation	Inertia	Bond Angles	Confor-mations	Segmental hops	Phys. Cluster	Bundle	
1000	0.008	0.48	21.3	1773	51100	57600	
2000	0.008	0.34	16.7	3257	46600	42100	
5000	0.008	0.42	16.4	895	33200	25500	
10000	0.008	0.48	19.1	765	21500	20300	QUICK
20000	0.008	0.38	11.4	837	19900	23800	
50000	0.008	0.30	7.6	2079	N.D.	N.D.	
100000	0.008	0.34	10.6	2071	N.D.	N.D.	SLOW
undef	0.008	0.46	9.8	N.D.	N.D.	N.D.	

deformation rate ($\dot{\gamma}$).

$$Wi = \frac{F(elastic)}{F(viscous)} = \dot{\gamma}\tau \tag{S11}$$

Table S3. Weissenberg numbers for various relaxation processes at different stretching rates; (v)- viscous; (e) -elastic; (ve)-viscoelastic process

Deformation rate [ps^{-1}]	Confor-mations	Segmental hops	Phys. Cluster	Bundle
10^{-3}	0.0213	1.773	51.1	57.6
$5 \cdot 10^{-4}$	0.0084	1.629	23.3	21.1
$2 \cdot 10^{-4}$	0.0033	0.179	6.6	5.10
10^{-4}	0.0019	0.076	2.25	2.03
$5 \cdot 10^{-5}$	0.0006	0.042	1.00	1.19
$2 \cdot 10^{-5}$	0.0002	0.042	N.D.	N.D.
10^{-5}	0.0001	0.021	N.D.	N.D.

REFERENCES

Barber, C. B., Dobkin, D. P., and Huhdanpaa, H. (1996). The quickhull algorithm for convex hulls. ACM Transactions on Mathematical Software 22, 469–483. doi:10.1145/235815.235821

Essmann, U., Perera, L., Berkowitz, M. L., Darden, T., Lee, H., and Pedersen, L. G. (1995-11-15). A smooth particle mesh ewald method. The Journal of Chemical Physics 103, 8577–8593. doi:10.1063/1.470117

Miyamoto, S. and Kollman, P. A. (1992). Molecular dynamics studies of calixspherand complexes with alkali metal cations. Journal of the American Chemical Society 114, 3668–3674. doi:10.1021/ja00036a015

Zidek, J., Milchev, A., Jancar, J., and Vilgis, T. A. (2016). Deformation-induced damage and recovery in model hydrogels – a molecular dynamics simulation. Journal of the Mechanics and Physics of Solids 94, 372–387. doi:10.1016/j.jmps.2016.05.013

Zidek, J., Milchev, A., Jancar, J., and Vilgis, T. A. (2017). Dynamic mechanical response of hybrid physical covalent networks – molecular dynamics simulation. Macromolecular Symposia 373, 1600147–. doi:10.1002/masy.201600147
